# Complex Interventions for a Complex System? Using Systems Thinking to Explore Ways to Address Unhealthy Commodity Industry Influence on Public Health Policy

**DOI:** 10.34172/ijhpm.2024.8033

**Published:** 2024-04-07

**Authors:** Adam Bertscher, Britta Katharina Matthes, James Nobles, Anna B. Gilmore, Krista Bondy, Amber van den Akker, Sarah Dance, Michael Bloomfield, Mateusz Zatoński

**Affiliations:** ^1^Department of Social and Policy Sciences, Faculty of Humanities & Social Sciences, University of Bath, Bath, UK.; ^2^Department of Health, Faculty of Humanities & Social Sciences, University of Bath, Bath, UK.; ^3^Centre of Active Lifestyles, Leeds Beckett University, Leeds, UK.; ^4^School of Management, Marketing, Business & Society, University of Bath, Bath, UK.; ^5^Department of Psychology, Faculty of Humanities & Social Sciences, University of Bath, Bath, UK.

**Keywords:** Systems Thinking Interventions, Complex Problem, Participatory Research, Unhealthy Commodities, Industry Influence, Commercial Determinants of Health

## Abstract

**Background:** Interventions are needed to prevent and mitigate unhealthy commodity industry (UCI) influence on public health policy. Whilst literature on interventions is emerging, current conceptualisations remain incomplete as they lack considerations of the wider systemic complexities surrounding UCI influence, which may limit intervention effectiveness. This study applies systems thinking as a theoretical lens to help identify and explore how possible interventions relate to one another in the systems in which they are embedded. Related challenges to addressing UCI influence on policy, and actions to support interventions, were also explored.

**Methods:** Online participatory workshops were conducted with stakeholders with expertise in UCIs. A systems map, depicting five pathways to UCI influence, and the Action Scales Model were used to help participants identify interventions and guide discussions. Codebook thematic analysis was used to analyse the data.

**Results:** Fifty-two stakeholders participated in 23 workshops. Participants identified 27 diverse, interconnected and interdependent interventions corresponding to the systems map’s pathways that reduce the ability of UCIs to influence policy. These include, for example, reform policy financing; regulate public-private partnerships (PPPs); reform science governance and funding; frame and reframe the narrative, challenge neoliberalism and gross domestic product (GDP) growth; leverage human rights; change practices on multistakeholder governance; and reform policy consultation and deliberation processes. Participants also identified four potential key challenges to interventions (ie, difficult to implement or achieve; partially formulated; exploited or misused; requires tailoring for context), and four key actions to help support intervention delivery (ie, coordinate and cooperate with stakeholders; invest in civil society; create a social movement; nurture leadership).

**Conclusion:** A systems thinking lens revealed the theoretical interdependence between disparate and heterogenous interventions. This suggests that to be effective, interventions need to align, work collectively, and be applied synchronously to different parts of the system, including multiple levels of governance. Importantly, these interventions need to be supported by intermediary actions to be achieved. Urgent action is now required to strengthen healthy alliances and implement interventions.

## Background

**Key Messages**

**Implications for policy makers**

- A systems thinking theoretical lens revealed that there is no single “solution” or “panacea,” but rather a disparate and heterogenous range of interventions that could be advanced to prevent and mitigate unhealthy commodity industry (UCI) influence on public health policy at multiple levels of governance.
- To effectively address UCI influence on public health policy, it is vital that stakeholders work collectively in healthy alliances at multiple levels of governance to coordinate and synergise their interventions across the system.
- Interventions to manage UCI influence on public health policy should be applied beyond policy-making processes and explicitly include other parts of the system, including at national and international levels, such as reforming corporate ownership, management, judicial proceedings, and Investor-State Dispute Settlement (ISDS) processes.
- Even when focusing on specific industries, public health advocates should actively consider the broader, generic issues of democratic governance and political, social, and economic reforms required to address corporate influence.

**Implications for the public**

The public should be concerned about how unhealthy commodity industries (UCIs) use their power to influence decision-making in ways that undermine public health, human rights, and democratic governance. This research shows that there is a wide range of interventions the public could support to prevent and mitigate UCIs from influencing public health policy. These interventions not only involve reforms to governance and policy-making processes, but also changes to social norms, and how we grow our economies and undertake business and science. Interventions need to work together and be applied across the system, including at multiple levels of governance, to help us achieve a healthier, fairer, and more democratic society. To achieve change, public support for these interventions can come in many forms, including active public engagement and participation in policy-making processes, raising awareness about corporate harms, and denormalising corporate involvement in decision-making processes that negatively impact the quality of our lives.

A core issue within the commercial determinants of health (CDoH) is the use of political practices by powerful industries to gain preferential treatment, or prevent, favourably shape, circumvent, and undermine public health policy.^1,2^ A group of these powerful industries that produce and sell “unhealthy commodities,” include tobacco, alcohol, and ultra-processed food. These commodities are major risk factors for non-communicable diseases^3,4^ and cause at least 14.5 million deaths per year globally.^1^ Other unhealthy commodities include fossil fuels, firearm and gambling industries, of which also pose substantial public health burdens.^5,6^

 A large body of research shows that these different unhealthy commodity industries (UCIs) consistently influence public health policy-making processes. For example, they engage in lobbying, shaping evidence by funding specific research agendas, creating front groups to advocate for industry-favourable positions, and use various arguments to frame debates around health policy, for instance, to place the burden of responsibility with individual’s behaviour or by arguing that regulation will likely fail.^2,7-15^

 Interventions are needed to restrain UCI power^16,17^ and prevent and mitigate UCI influence on public health policy to enable effective regulation for unhealthy commodities.^7,18-21^ Recently public health literature has began to categorise or discuss interventions that cut across UCIs, shifting away from industry-specific interventions.^18,21-35^ For example, a scoping review^18^ catalogues various governance mechanisms that can help address UCI influence, namely transparency and disclosure of industry practices and conflicts of interest (CoIs) (eg, lobbying registers); identification, monitoring, and education about industry’s harmful practices (eg, inter-departmental training or governmental administrative circulars); management of interactions and of CoI (eg, prohibitions on gifts and donations to decision-makers); and prohibition of interactions with industry (eg, excluding industries from policy consultations). To date, many of these interventions were implemented in relation only to the tobacco industry because states who ratified the World Health Organization (WHO) Framework Convention on Tobacco Control (FCTC) were obligated to implement interventions in relation to Article 5.3. which required them to protect public health policies from commercial and other vested interests of the tobacco industry.^18,36^ Other scholars discuss “solutions” to CDoH that counter UCI power more generally, such as alternatives to investing and economic growth.^1,35^ Other proposed strategies to enable public health actors to counter industry influence include “protect[ing] public health advocates from industry threats”^21^ or “expand[ing] public health training and coalitions.”^21^

 Despite this growing literature, there remain important gaps in our understanding of interventions to address UCI influence. Firstly, there is a lack of a broader list of potential interventions targeting different systemic pathways to UCI influence. Secondly, there is a lack of an understanding of the cross-cutting challenges to interventions and actions needed to achieve them. Thirdly, literature on interventions does not consider the complexities and systems nature of such UCI influence,^37^ such as how interventions are interconnected and interdependent or how industry adapts to them.^12,38-40^

 Research suggests that UCI influence on public health policy could be characterised as part of a complex system^41-43^ – ie, a system that adapts over time in response to changes.^44^ System thinking is a way of understanding and conceptualising complex problems, often using participatory methods.^45-52^ It would therefore be a suitable approach to identify and explore potential interventions aiming to address UCI influence on public health policy.^1,41-43^ A systems thinking approach can consolidate these proposed interventions coherently within one paper, which is — to our knowledge — the first time this has been done, thereby showing the range of potential interventions for stakeholders not familiar with them.

 Our previous research mapped five interconnected pathways which UCIs use to influence policy (Table 1), namely, (1) directly accessing public sector decision-makers; (2) creating confusion and doubt about policy decisions; (3) prioritising corporate profits and growth; (4) leveraging legal and dispute settlement processes; and (5) leveraging policy-making norms, rules, and processes.^37^ Such maps can be used to help identify potential interventions. Therefore, building on this systems map, the current study sought to explore:

1. What do stakeholders perceive as possible interventions to prevent and mitigate UCI influence on public health policy in different parts of the system?
2. What are the associated challenges and actions to help achieve these interventions?
3. What is the potential interconnectivity and interdependence between the identified interventions?

**Table 1:** Pathways Unhealthy Commodity Industries Use to Influence Public Health Policy

Pathway	Description^a^
1. Directly accessing public sector decision-makers	The extent to which public sector decision-makers (policy-makers, civil servants, public officials) can be directly accessed by industry actors which can be formal (eg, being part of a policy committee) and informal (eg, through interpersonal relationships).
2. Creating confusion and doubt about policy decisions	The extent to which decision-makers, and the public are confused about whether the proposed policy is needed and will lead to public value.
3. Corporate prioritisation of commercial profits and growth	The extent to which corporations prioritise their own profits and growth above other economic costs associated with consuming unhealthy commodities (or other societal values, such as health, well-being, human rights, and the natural environment).
4. Leveraging legal and dispute settlement processes	The extent to which industry leverages legal and dispute settlement processes, for example, by bringing or threatening to bring litigation against governments to prevent, undermine, or reverse public health policy.^b^
5. Leveraging policy-making norms, rules, and processes	The extent to which industry leverages national and international policy-making norms, rules and processes that favour its participation in policy-making.

^a^ Descriptions are from Bertscher et al.^37^ ^b^ This path includes government obligations to follow international trade and investment agreements.

## Methods

###  Study Design

 We conducted a series of online participatory workshops^46^ to identify and discuss interventions to achieve change and understand the potential theoretical interconnectivity and interdependence between interventions. Participatory workshops are a well-accepted method in systems science.^47,53-57^

###  Participant Recruitment

 We purposively recruited participants with diverse experiences and backgrounds (ie, academia, civil society, former public office, and global governance organisations), and different expertise areas, geographical regions and policy levels (See Table 2). Potential participants were initially identified through literature reviews and authors’ networks and then through snowballing. Email invitations were sent to 83 participants, with 52 taking part. Sixteen were unresponsive and 15 declined to participate.

**Table 2:** Stakeholder List and Background

**Participant Background**		**Number of Participants**
Stakeholder group (participant reference codes)	Academia	26
	Civil society	20
	Former public official	4
	Global governance organisation	2
Expertise^a^	Ultra-processed foods	27
	Alcohol	24
	Tobacco	29
	CDoH and industry influence	38
	Economics	1
	International trade	4
	Policy-making	12
	Law	5
Geographical region of expertise^a^ (WHO regions)	African region	17
	Region of the Americas	17
	South-East Asian region	4
	European region	27
	Eastern Mediterranean region	1
	Western Pacific region	15

Abbreviations: CDoH, commercial determinants of health; WHO, World Health Organization. ^a^Participant expertise area (ie, tobacco, alcohol, etc) and geographical region of expertise (ie, African region, European region, etc) may fall within more than one category.

###  Workshops

 A total of 23 workshops were conducted between November 2021 to February 2022. Each workshop lasted between 60 to 120 minutes and had one to five participants in each. Based on their own experiences and knowledge, participants were asked to (*a*) brainstorm interventions which could address UCI influence, (*b*) consider general challenges associated with implementing interventions, and (*c*) identify ways to support intervention implementation.

 We used a recognised approach to structure the workshops, adapting systems mapping activities from Scriptapedia.^58^ The systems map^37^ provided a starting point for identifying potential interventions to address UCI influence; workshops allowed for participants to create a uniform baseline understanding of the system and critically engage with the systems map. Participants were sent the map and information prior to the workshop to allow familiarisation with its content. The Action Scales Model^53^ was used as a conceptual tool to help facilitate group discussions by challenging participants to consider the underlying drivers and conditions which enable UCI influence. This model proposed four levels to intervene in a system, namely events, structures, goals and beliefs. According to the model, intervening in the deeper levels (ie, beliefs) will provide greater potential for systems change.^53^ During the workshops, the facilitator (AB) guided discussions around the systems map’s themes using an online whiteboard, inviting participants to insert their written comments about interventions. Given time constraints, each workshop focused on aspects of the map where participants had particular expertise, although participants were still able to review, comment on, and discuss the whole map.

 Two pilot workshops were conducted which led to minor adaptations in the workshop structure. All workshops were conducted on Microsoft Teams, and were recorded and transcribed. A notetaker (AVDA or SD) was present in each workshop to capture key discussion points and researchers’ reflections, and to provide technical assistance when required.

###  Analysis

 Codebook Thematic Analysis^59^ was conducted on workshop transcripts, notes and participants’ written comments. A preliminary codebook of interventions was developed based on familiarisation and pre-reading of the data and was further refined during the coding process. Each intervention was also coded to a systems map theme. Challenges and actions to help achieve interventions, as well as interconnectivity and interdependence between interventions were identified inductively.

 Pilot coding was conducted on 25% of the workshop data by AB and BKM, who met to discuss and refine the codebook. After pilot coding, AB and BKM independently coded 50% of the remaining data. AB then single coded the remaining 50%. As coding continued, additional codes were developed. Similar codes were then grouped and synthesised to form categories for interventions, actions and challenges. During this process, potential interconnectivity and interdependence was identified by coding for their overlaps and relationships. Analysis continued in an iterative manner until all workshop data were analysed. AB and BKM regularly met to discuss the results and reach consensus on any divergent views.

## Results

**Figure 1:**
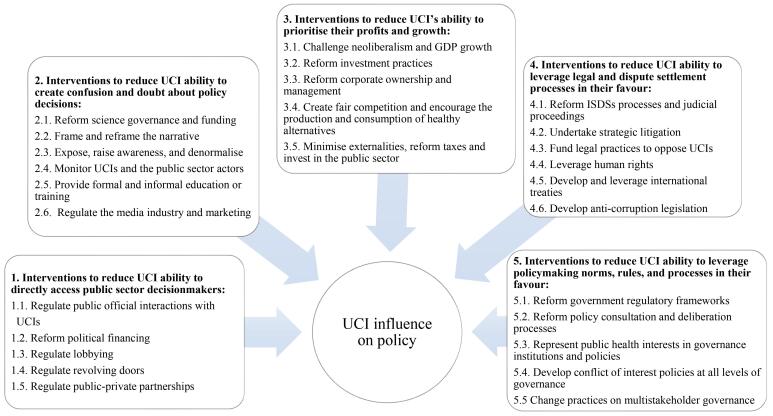
Interventions Identified to Address UCI Influence on Public Health Policy Across the System. Abbreviations: UCI, unhealthy commodity industry; GDP, gross domestic product; ISDSs, Investor-State Dispute Settlements.

 Given the use of the systems map, interventions were grouped according to relevance for each of the five pathways as shown in Figure 1. The following sections presents the interventions categories, associated actions, and potential challenges to interventions and potential interconnectivity and interdependence between them, as indicated by italicised references below. Supplementary file 1 includes participant references and illustrative quotes corresponding to each section.

###  Interventions

####  1. Interventions to Reduce UCI Ability to Directly Access Public Sector Decision-Makers

####  1.1. Regulate Public Official Interactions With UCIs

 Participants proposed that governments should develop clearly defined mandatory rules of engagement or codes of conduct for public officials by detailing how they should be permitted to interact with representatives of UCIs. Participants suggested enforcement and sanctions for violations of such rules or codes, and improved enforcement and implementation of WHO FCTC Article 5.3. for representatives of all government departments, not just departments for health (*intervention 2.4. monitor UCIs and public sector actors*).

####  1.2. Reform Political Financing

 Participants recommended enforced restrictions on political donations from UCIs to politicians, political parties, or election campaigns, and real-time reporting and disclosure of all such political financing (*intervention 2.4. monitor UCIs and public sector actors*).

####  1.3. Regulate Lobbying

 Participants recommended a complete prohibition of UCIs from lobbying by developing publicly accessible lobbying registers consisting of mandatory disclosures, including of gifts. This should involve monitoring and the timely reporting of all lobbying practices directed at public officials, including judicial officials. Participants also mentioned that this this type of regulation should be balanced with the need for policy-makers to carry out their duties and interact with the necessary stakeholders. Importantly, it is essential to have adequate oversight and enforcement procedures for lobbying registers, and mandatory disclosures of lobbyists’ CoI (*interventions 2.4. monitor UCIs and public sector actors*; and *5.4. develop CoI policies at all levels of governance*).

####  1.4. Regulate Revolving Doors

 Participants suggested that revolving door practices should be prohibited by restricting the number of years before public officials can work for the private sector, or vice versa. This includes prohibiting public sector officials from having second jobs. Participants suggested oversight and enforcement of revolving doors regulation and removal of public officials implicated in such practices (*intervention 2.4. monitor UCIs and public sector actors*).

####  1.5. Regulate Public-Private Partnerships 

 Standards for managing or forming public-private partnerships (PPPs) at all levels of governance should be developed, including financial transparency, and monitoring PPPs (*intervention 2.4. monitor UCIs and the public sector actors*). This should include mandatory disclosure of CoI for those involved in PPPs, and prohibition on involvement in PPPs if CoI are found (*intervention 5.4. develop CoI policies at all levels of governance*). Moreover, independent evaluations of PPPs should be conducted (*intervention 2.1. reform science governance and funding*).

####  2. Interventions to Reduce UCI Ability to Create Confusion and Doubt About Policy Decisions

####  2.1. Reform Science Governance and Funding

 Participants recommended that governments establish independent public oversight bodies, such as public health agencies, to monitor and evaluate public health evidence to a benchmark standard for policy-making purposes. Such governing institutions could decide on consequences for the presence of CoI in research submitted for policy-making purposes and should monitor the progress and goals for regulating unhealthy commodities, whilst being reliably and adequately funded (*intervention 3.5. minimise externalities, reform taxes, and invest in the public sector)*. Participants also suggested that civil society or independent academics should be required in the oversight of industry research compliance.

 There should be mandatory disclosure of CoI in private sector research submitted for policy-making purposes, including disclosure of funding of researchers and think tanks. Universities, professional associations and academic journals should develop CoI policies (*intervention 5.4. develop CoI policies at all levels of governance*). Alternative funding systems and rules for research on unhealthy commodities should be established that reduces UCI ability to influence research by adequately public funding of science on unhealthy commodities or restricting UCIs from funding academics (*intervention 3.5. minimise externalities, reform taxes, and invest in the public sector*).

####  2.2. Frame and Reframe the Narrative

 Participants proposed numerous ways to frame and reframe narratives around unhealthy commodities and UCI influence on public health policy. This would include framing *public health issues*: as a matter of social justice; those suffering from non-communicable diseases are victims of UCI practices; and UCIs make profit from “death.” Participants also suggested framing *UCI influence *as a matter of corruption, social justice, and democratic principles violations (*intervention 4.4. leverage human rights*); and framing *unhealthy commodity consumption* as socially unacceptable, part of the climate change agenda, and not as the result of individuals’ choice or responsibility, but due to UCI influence on individuals’ behaviours.

 Participants also suggestedframing that: corporations should be accountable for their actions analogous to natural persons (*intervention 3.3. reform corporate ownership and management*); UCIs have a CoI in public health policy (*intervention 5.4. develop CoI policies at all levels of governance*); UCI regulation would create a healthier society providing economic and business benefits (*intervention 3.1. challenge neoliberalism and gross domestic product [GDP] growth*); it is inappropriate for UCIs to make scientific claims about the medical or public health impacts of their products; and UCIs do not have moral autonomy or authority.

 Participants suggested challenging the frame that UCIs are ‘good citizens’ or benevolent actors, and industry self-regulation and PPPs are altruistic initiatives. Lastly, participants suggested targeted framing or reframing the narrative at decision-makers who have vested interests in UCIs or who support neoliberal policies.

####  2.3. Expose, Raise Awareness, and Denormalise

 Participants suggested that UCI practices should be exposed to help raise awareness, and thus denormalise unhealthy commodities and UCIs.

 Firstly, participants recommended *exposing* the relationships between public officials, heads of UCIs, and UCI association representatives; overlaps of boards of directors between UCIs; issues with multistakeholderism and PPPs and corporate social responsibility (*intervention 1.5. regulate public-private partnerships*). Moreover, participants recommended exposing how UCIs influence evidence; harms caused by UCI products; consumer misinformation; the use of UCI front groups and funded organisations; and issues of poor transparency in policy-making.

 Secondly, one could raise awareness of UCI arguments to “inoculate” or prepare the public for them and to foster public outrage about issues of UCI influence to help change public opinion and demand government to act (*intervention 2.2. frame and reframe the narrative*).

 Thirdly, participants suggested denormalising UCI products and practices by exposing UCI practices, as suggested above, by: working with journalists and using online and offline media, including social media; reporting on decision-makers’ interactions with UCIs, their CoI, and corrupt practices (*intervention 2.4. monitor UCIs and public sector actors*); using public relations strategies, such as leveraging medical professionals and experts to convey public health messages; sharing personal experiences of UCIs attempts to influence policy; translating academic research into communication products that resonate with policy-makers and consumers; and targeting decision-makers by communicating the impact of UCIs on health.

####  2.4. Monitor UCIs and Public Sector Actors

 Participants suggested establishing civil society watchdog groups to monitor UCI: political practices (*interventions 1.3. regulate lobbying; 1.4. regulate revolving doors; and 1.5. regulate public-private partnerships*); influence on science (*intervention 2.1. reform science governance and funding*); financing activities; mergers and acquisitions, global corporate tax payments; and violations of, and inappropriate, marketing practices. Stop Tobacco Organization and Products (STOP) was noted as good example of industry monitoring. There should also be monitoring of decision-makers’ voting behaviours in parliament, and legislation should be developed to institutionalise the role of civil society in monitoring the government and private sector.

 To adequately monitor, there should be improvements in transparency for decision-making concerning unhealthy commodities. Participants proposed improving freedom-of-information request processes and applying them to public bodies that receive public funding or may have a regulatory role, such as advertising standards authorities; and there should be mandatory and standardised disclosures for UCIs on sales, marketing and operations data.

####  2.5. Provide Formal and Informal Education or Training

 Participants recommended formal and informal education or training be developed for issues of unhealthy commodities and UCI influence on policy, for example, in school and university curricula, especially in business schools. Schools should include media literacy so that people are critical about UCI advertising practices.

 Training should be targeted at policy-makers at national and international levels, as well as members of civil society, university staff, and judicial officers not familiar with UCI influencing practices (*intervention 3.5. minimise externalities, reform taxes, and invest in the public sector*). It should include the need for public health policies, such as for UCI marketing practices (*intervention 2.2. frame and reframe the narrative*). Academics and civil society groups who are knowledgeable of UCI influencing practices should work with the media to develop public education campaigns. They should also develop public education on the issues around processes that enables UCI influence (*intervention 2.3. expose, raise awareness, and denormalise*), such as investor-state dispute settlement (ISDS) processes (*intervention 4.1. reform Investor-State Dispute Settlements processes and judicial proceedings*).

####  2.6. Regulate the Media Industry and Marketing

 Media companies should be mandated to disclose CoI of lobbyists and industry-funded research in media coverage (*intervention 2.1. reform science governance and funding; and 5.4. develop CoI policies at all levels of governance*). Media companies should also be prohibited from providing platforms to organisations who fail to disclose funding sources.

 There should be restrictions on marketing of UCI products online and offline, particularly to children. Participants noted France’s 1991 Loi Évin as a good example of marketing regulations as it prohibits alcohol and tobacco advertising on TV or cinema, and requires a health warming accompanying any permitted alcohol advertising. However, researchers have recently argued that Loi Évin may need to be updated to remain fit for purpose specifically in the digital age. Regulations should also restrict the amount UCIs can spend on advertising, and there should be adequate enforcement mechanisms for such regulations (*intervention 3.5. minimise externalities, reform taxes, and invest in the public sector*).

####  3. Interventions to Reduce UCI ability to Prioritise Their Profits and Growth

####  3.1. Challenge neoliberalism and GDP growth

 Participants proposed challenging the fundamental assumptions of neoliberalism, and adopting degrowth or well-being economies by applying alternative measures of economic growth that embed public health, equitable distribution goals, and account for the depletion of the natural environment (*intervention 3.5. minimise externalities, reform taxes, and invest in the public sector*). For example, Bhutan’s Gross National Happiness Index, which seeks to achieve a level of happiness and well-being in their development goals. Participants also suggested incentivising corporations to aim for positive impact on the environment and social, health and employee wellness, and develop greater benefit sharing mechanisms and debt forgiveness in international development practices (*intervention 3.2. reform investment practices*).

####  3.2. Reform Investment Practices

 International standards on socially responsible investing or environmental, social, and governance investing should be developed for individuals or insurance companies (*intervention 3.1. challenge neoliberalism and GDP growth*). Participants suggested that investors should undertake social impact investing and invest in impact weighted accounts, and governments should invest in small and medium enterprises instead of large corporations.

####  3.3. Reform Corporate Ownership and Management

 Corporate charters and corporate legal entities should be changed to include social and environmental impacts as corporate goals, such as Benefit Corporations (*intervention 3.2. reform investment practices*). Rules on corporate fiduciary duties should change to include social and environmental stakeholders, remove corporate limited liability, and mandate direct liability. This would allow for corporate owners or management to be liable for company wrongdoings, such as for violations of human rights and environmental standards (*4.2. undertake strategic litigation; 4.4. leverage human rights; 4.5. develop and leverage international treaties*). If significant corporate harm is found, there could be judicial dissolution of, or restricting, the corporate entity in certain jurisdictions.

 Alternative forms of business structures should be developed. This may include co-operatives that mandate worker participation in company decisions or establishing government-owned manufacturing and/or retailing companies for unhealthy commodities, for example, modelled on Canada’s Provincial Liquor Crown Companies or the Vinmonopolet (ie, Norwegian government-owned alcoholic beverage retailer). Moreover, there could be remunicipalisation of some public services and mandating diversification of corporate boards (*intervention 3.1. challenge neoliberalism and GDP growth*).

####  3.4. Create fair Competition and Encourage the Production and Consumption of Healthy Alternatives 

 Participants suggested that UCIs and media industry monopolies should be broken up. Governments could support newcomers of healthier products or services into the market to compete with UCI incumbents. For example, they could support smaller local businesses changing rules on subsidies to UCIs, or providing healthier alternatives to consumers, through food aid programs or universal basic income (*intervention 3.1. challenge neoliberalism and GDP growth*).

####  3.5. Minimise Externalities, Reform Taxes, and Invest in the Public Sector

 Governments should minimise externalities through mandating true cost accounting, and introducing “polluter pays principle” by taxing unhealthy commodities. Importantly, there should be: improved enforcement, collection, monitoring, and oversight of existing tax laws; the elimination of corporate tax avoidance; the closing of loopholes for tax havens, evasion, and deductibles; and tighten of controls on corporate tax liabilities. International regulations for tax could be developed, such as a global consensus for corporate tax baseline through the United Nations (UN) (*intervention 4.5. develop and leverage international treaties*). Participants proposed giving Departments for Health control over taxes that impact public health, such as setting or earmarking taxes on unhealthy commodities to use for public health services, or establishing independent health promotion foundations, as seen with the Thai Health Promotion Foundation (*intervention 2.1. reform science governance and funding; and 5.3. represent public health interests in governance institutions and policies*).

 Governments should institute progressive and redistributive taxation to adequately fund public services and international governance institutions, so that they do not need to rely on PPPs or private sector donations. This would strengthen public institutions to better protect and promote public health interests (*intervention 1.5. regulate public-private partnerships*).

 Governments should provide funding to implement regulations for UCIs, including the funding of public regulatory bodies to enforce regulations and provide counter advertising for unhealthy commodities or education campaigns explaining how health policies will be effective. Such investments could also develop the skills of public sector officials so that they understand how UCIs influence policy (*intervention 2.5. provide formal and informal education or training*).

####  4. Interventions to Reduce UCI Ability to Leverage Legal and Dispute Settlement Processes in Their Favour 

####  4.1. Reform Investor-State Dispute Settlements Processes and Judicial Proceedings

 To help curb UCI ability to leverage legal and dispute settlement processes, participants suggested reforming ISDS processes by amending World Trade Organization (WTO) rules. ISDS processes should be changed to allow for the establishment of mechanisms for some forms of arbitration to be dealt with in domestic courts; reduce arbitration costs for governments in domestic courts; mandate greater arbitrator diversification in ISDS courts; and cap compensation in court decisions, for example, based on a country’s GDP or income level.

 Participants also suggested developing stronger public health protections for UCIs in WTO rules, such as public health clauses, or exempting public health protections from litigation; the 2001 WTO Doha declaration, which sought to balance protecting intellectual property rights and ensuring access to essential medicines, was noted as a good example of this (*intervention 4.1. reform Investor-State Dispute Settlements processes and judicial proceedings; 5.3. represent public health interests in governance institutions and policies*).

 Domestic resources should be mobilised through the taxation of externalities to fund government legal defence in ISDS processes (*intervention 3.5. minimise externalities, reform taxes, and invest in the public sector*). There should be mandatory disclosure of CoI for judicial officials and arbitrators, and recusals in ISDS proceedings where CoI are found (*intervention 5.4. develop CoI policies at all levels of governance*).

####  4.2. Undertake Strategic Litigation

 Participants suggested bringing strategic litigation against UCIs or governments by leveraging existing consumer law, especially on misleading information. Making changes to the status of corporate legal entity, such as limited liability, would support strategic litigation (*intervention 3.3. reform corporate ownership and management*).

 “Victims” could bring cases against UCIs for their attempts to influence policy and encouraging the consumption of unhealthy commodities (*intervention 2.3. frame and reframe the narrative*). Litigation or disciplinary hearings could also be used to enforce policies such as, CoI policies in decision-making (*intervention 5.4. develop CoI policies at all levels of governance*). There should also be international cooperation between governments and civil society to support each other when UCIs litigates against government.

####  4.3. Fund Legal Practices to Oppose UCIs

 A WHO fund could be created to provide legal support for governments undertaking legal action against UCIs. A public interest legal fund could assist whistle-blowers and governments in defending against litigation by UCIs or to engage in arbitration processes (*intervention 4.6. develop anti-corruption legislation*). Participants suggested mobilising domestic funding, such as taxes, to fund civil society legal actions, and proposed that philanthropies who fund industry monitoring organisations could also fund organisations’ legal activities (*intervention 3.5. minimise externalities, reform taxes, and invest in the public sector*).

####  4.4. Leverage Human Rights

 Legislation should be developed mandating human rights and environmental due diligence for supply chains, and the direct liability for violations of human rights standards by corporations at the national and international levels *(intervention 3.3. reform corporate ownership and management*). Civil society and governments should utilise UN reporting mechanisms for human rights abuses by business (*intervention 2.4. monitor UCIs and public sector actors*).

 Government’s human rights obligations should be leveraged, such as the right to health, enjoy the benefits of scientific progress, access healthy environments, and public participation. This would help to prioritise social and public health goals in national and global level decision-making and encourage greater civil society participation in policy processes (*intervention 2.2. frame and reframe the narrative*; and* 5.2. reform policy consultation and deliberation processes*). Human rights goals should also be enshrined in international trade and investment agreements (ITIAs) (*intervention 4.5. develop and leverage international treaties*).

####  4.5. Develop and Leverage International Treaties

 International treaties comparable to WHO FCTC for each unhealthy commodity should be developed which would include an Article 5.3. equivalent. Participants also suggested developing other treaties, such as a Framework Convention on Global Health, based on the right to health, international regulations for corporate tax baseline, or an international treaty on business and human rights with strong built-in enforcement mechanisms (*intervention 4.4. leverage human rights*). Such treaties could be used as counterweights to ITIAs, especially in ISDSs. Implementation and adherence to any existing and future treaties could be improved through close coordination between international governance institutions and national policy-makers, especially the WHO FCTC Article 5.3. (*intervention 2.5. minimise externalities, reform taxes, and invest in the public sector; and 5.3. represent public health interests in governance institutions and policies*).

####  4.6. Develop Anti-corruption Legislation

 Participants suggested developing anti-corruption legislation to make political practices UCI use to influence policy-makers a serious criminal offense. Such legislation would cover UCI lobbying, political financing, revolving door practices, transgressions regarding CoI, and bribery by UCIs, as well as provide protections for whistle-blowers* (interventions 1.2. reform political financing; 1.3. regulate lobbying; 1.4. regulate revolving doors; 2.3. expose, raise awareness, and denormalise; 2.4. monitor UCIs and public sector actors; and 5.4. develop CoI policies at all levels of governance).*

####  5. Interventions to Reduce UCI Ability to Leverage Policy-Making Norms, Rules, and Processes in Their Favour

####  5.1. Reform Government Regulatory Frameworks

 Health impact assessments (HIAs) should be mandated for national and international policy-making, including for ITIA and industry self-regulation. HIAs should include the economic costs of public health problems and the benefits of regulation for populations’ social life, health, and well-being *(intervention 3.5. minimise externalities, reform taxes, and invest in the public sector)*. Finland’s Health in All Policy approach was noted as a good example for considering the public health implications of policy decisions. In addition, participant suggested that accepted standards for the assessments of public health risks of products should be developed and introduced for regulating UCIs (*intervention 3.1. challenge neoliberalism and GDP growth*).

####  5.2. Reform Policy Consultation and Deliberation Processes 

 Participants recommended formal rules, such as parliamentary procedures, on how public officials should engage with UCIs in policy consultations and deliberations (*intervention 1.1. regulate public official interactions with UCIs*). Participants suggested requirements for public and civil society representation or participation in policy-making, for example, deliberative policy-making processes at national and international levels, including in ITIA negotiations, the WTO, and global governance organisations, namely the World Bank and UN agencies (*intervention 5.3. represent public health interests in governance institutions and policies*). A participant noted these approaches have successfully applied to climate policy. Standards should be developed for the inclusion of evidence in policy consultation and deliberation processes (*intervention 2.1. reform science governance and funding*).

####  5.3. Represent Public Health Interests in Governance Institutions and Policies

 Public health actors should be represented in trade regulation activities, including ITIA negotiations (*intervention 4.5. develop and leverage international treaties*). More power could be given to health ministries, such as ensuring that departments for health lead on policies with health implications (*intervention 3.1. challenge neoliberalism and GDP growth*). Governments could also develop strategies to achieve policy coherence, such as instituting inter-ministerial committees for public health issues, reflected in Finland’s Health in All Policy Approach noted above (*5.1. reform government regulatory frameworks*).

####  5.4. Develop CoI Policies at All Levels of Governance

 Rules for personal, institutional or financial CoIs should be developed for national governments and international governance organisations, including decision-maker dealings with the private sector, declaration of owning UCI stocks and shares by policy-makers, and the removal or recusal of public officials from working on policy issues if CoIs are found (*intervention 2.4. monitor UCIs and public sector actors*). Participants noted that generally CoI policies were not well understood or conceptualised in governing and research institutions. There should be mandatory disclosure of CoI for policy actors in policy consultations or deliberations, such as industry representatives or third parties, including consulting firms or civil society groups (*interventions 1.3. regulate lobbying; 1.4. regulate revolving doors; and 1.5. regulate public-private partnerships*).

 For individuals or organisations representing UCIs (eg, corporate officials, producers, and marketers) that have a personal, institutional or financial CoI in a policy area, there should be mandatory restrictions on their participation in formal or informal governmental committees or policy discussions and consultations, and there should be sanctions and enforcement for violations of CoI rules. Governments should create a governing body to decide whether a CoI exists in a particular instance, monitor public officials for CoIs, and provide capacity building for decision-makers around issues of CoI (*intervention 2.1. reform science governance and funding; *and *2.5. provide formal and informal education or training*).

####  5.5. Change Practices on Multistakeholder Governance

 Participants suggested changing practices on multistakeholder governance that promote UCIs as legitimate governance actors involved in policy-making (*intervention 2.3. expose, raise awareness, and denormalise*). Advocates should seek to counteract UCI involvement in policy-making processes, particularly at international governance institutions, such as the WHO (*intervention 2.2. frame and reframe the narrative*).

###  Potential Challenges to Advance Interventions

 The following are potential cross-cutting challenges to advance interventions (See Table 3 for their interconnectivity and interdependence).

**Table 3:** Interconnectivity and Interdependence Between Interventions, Actions or Challenges

**Systems Map Themes**	**Intervention Category**	**Potential Interconnectivity and Interdependencies With Interventions, Actions or Challenges**
1. Interventions to reduce UCI ability to directly access public sector decision-makers	1.1. Regulate public official interactions with UCIs	→ Intervention 2.4. Monitor UCIs and public sector actors ← Challenge 2. Partially formulated or implemented interventions
	1.2. Reform political financing	→ Intervention 2.4. Monitor UCIs and public sector actors → Intervention 5.4. Develop CoI policies at all levels of governance ← Challenge 2. Partially formulated or implemented interventions
	1.3. Regulate lobbying	→ Intervention 2.4. Monitor UCIs and public sector actors → Intervention 5.4. Develop CoI policies at all levels of governance ← Challenge 2. Partially formulated or implemented interventions
	1.4. Regulate revolving doors	→ Intervention 2.4. Monitor UCIs and public sector actors ← Challenge 2. Partially formulated or implemented interventions
	1.5. Regulate PPPs	→ Intervention 2.1. Reform science governance and funding → Intervention 2.4. Monitor UCIs and the public sector actors → Intervention 5.4. Develop CoI policies at all levels of governance
2. Interventions to reduce UCI ability to create confusion and doubt about policy decisions	2.1. Reform science governance and funding	→ Intervention 3.5. Minimise externalities, reform taxes, and invest in the public sector → Intervention 5.4. Develop CoI policies at all levels of governance ← Action 1. Coordinate and cooperate between stakeholders ← Action 2. Invest in civil society
	2.2. Frame and reframe the narrative	→ Intervention 3.1. Challenge neoliberalism and GDP growth → Intervention 3.3. Reform corporate ownership and management → Intervention 4.4. Leverage human rights → Intervention 5.4. Develop CoI policies at all levels of governance
	2.3. Expose, raise awareness, and denormalise	→ Intervention 1.5. Regulate public-private partnerships → Intervention 2.2. Frame and reframe the narrative → Intervention 2.4. Monitor UCIs and public sector actors ← Action 1. Coordinate and cooperate between stakeholders ← Action 4. Nurture leadership
	2.4. Monitor UCIs and the public sector actors	→ Intervention 1.3. Regulate lobbying → Intervention 1.4. Regulate revolving doors → Intervention 1.5. Regulate public-private partnerships → Intervention 2.1. Reform science governance and funding ← Action 2. Invest in civil society
	2.5. Provide formal and informal education or training	→ Intervention 2.2. Frame and reframe the narrative → Intervention 2.3. Expose, raise awareness, and denormalise → Intervention 3.5. Minimise externalities, reform taxes, and invest in the public sector → Intervention 4.1. Reform ISDS processes and judicial proceedings ← Action 1. Coordinate and cooperate between stakeholders ← Action 2. Invest in civil society ← Action 4. Nurture leadership
	2.6. Regulate the media industry and marketing	→ Intervention 2.1. Reform science governance and funding → Intervention 3.5. Mminimise externalities, reform taxes, and invest in the public sector → Intervention 5.4. Develop CoI policies at all levels of governance ← Challenge 2. Partially formulated or implemented interventions
3. Interventions to reduce UCI ability to prioritise their profits and growth	3.1. Challenge neoliberalism and GDP growth	→ Intervention 3.2. Reform investment practices → Intervention 3.5. Minimise externalities, reform taxes, and invest in the public sector ← Challenge 1. Difficult to achieve or implement ← Action 3. Create a social movement
	3.2. Reform investment practices	→ Intervention 3.1. Challenge neoliberalism and GDP growth ← Challenge 3. Exploited or misused interventions
	3.3. Reform corporate ownership and management	→ Intervention 3.1. Challenge neoliberalism and GDP growth → Intervention 3.2. Reform investment practices → Intervention 4.2. Undertake strategic litigation → Intervention 4.4. Leverage human rights → Intervention 4.5. Develop and leverage international treaties ← Challenge 3. Exploited or misused interventions
	3.4. Create fair competition and encourage the production and consumption of healthy alternatives	→ Intervention 3.1. Challenge neoliberalism and GDP growth ← Challenge 4. Interventions require tailoring for context
	3.5. Minimise externalities, reform taxes and invest in the public sector	→ Intervention 1.5. Regulate public-private partnerships → Intervention 2.1. Reform science governance and funding → Intervention 2.5. Provide formal and informal education or training → Intervention 4.5. Develop and leverage international treaties → Intervention 5.3. Represent public health interests in governance institutions and policies ← Challenge 1. Difficult to achieve or implement
4. Interventions to reduce UCI ability to leverage legal and dispute settlement processes in their favour	4.1. Reform ISDS processes and judicial proceedings	→ Intervention 3.5. Minimise externalities, reform taxes, and invest in the public sector → Intervention 5.3. Represent public health interests in governance institutions and policies → Intervention 5.4. Develop CoI policies at all levels of governance ← Challenge 1. Difficult to achieve or implement
	4.2. Undertake strategic litigation	→ Intervention 2.3. Frame and reframe the narrative → Intervention 3.3. Reform corporate ownership and management → Intervention 5.4. Develop COI policies at all levels of governance ← Action 1. Coordinate and cooperate between stakeholders
	4.3. Fund legal practices to oppose UCIs	→ Intervention 3.5. Minimise externalities, reform taxes, and invest in the public sector → Intervention 4.6. Develop anti-corruption legislation
	4.4. Leverage human rights	→ Intervention 2.2. Frame and reframe the narrative → Intervention 2.4. Monitor UCIs and public sector actors → Intervention 3.3. Reform corporate ownership and management → Intervention 4.5. Develop and leverage international treaties → Intervention 5.2. Reform policy consultation and deliberation processes ← Action 2. Invest in civil society
	4.5. Develop and leverage international treaties	→ Intervention 2.5. Minimise externalities, reform taxes, and invest in the public sector → Intervention 4.4. Leverage human rights → Intervention 5.3. Represent public health interests in governance institutions and policies ← Challenge 1. Difficult to achieve or implement ← Action 1. Coordinate and cooperate between stakeholders
	4.6. Develop anti-corruption legislation	→ Intervention 1.2. Reform political financing → Intervention 1.3. Regulate lobbying → Intervention 1.4. Regulate revolving doors → Intervention 2.3. Expose, raise awareness, and denormalise → Intervention 2.4. Monitor UCIs and public sector actors ← Challenge 4. Interventions require tailoring for context
5. Interventions to reduce UCI ability to leverage policy-making norms, rules, and processes in their favour	5.1. Reform government regulatory frameworks	→ Intervention 3.1. Challenge neoliberalism and GDP growth → Intervention 3.5. Minimise externalities, reform taxes, and invest in the public sector
	5.2. Reform policy consultation and deliberation processes	→ Intervention 1.1. Regulate public official interactions with UCIs → Intervention 2.1. Reform science governance and funding → Intervention 5.3. Represent public health interests in governance institutions and policies ← Challenge 3. Exploited or misused interventions ← Action 2. Invest in civil society
	5.3. Represent public health interests in governance institutions and policies	→ Intervention 3.1. Challenge neoliberalism and GDP growth → Intervention 4.5. Develop and leverage international treaties → Intervention 5.1. Reform government regulatory frameworks ← Action 1. Coordinate and cooperate between stakeholders
	5.4. Develop COI policies at all levels of governance	→ Intervention 1.3. Regulate lobbying → Intervention 1.4. Regulate revolving doors → Intervention 1.5. Regulate public-private partnerships → Intervention 2.1. Reform science governance and funding → Intervention 2.4. Monitor UCIs and public sector actors → Intervention 2.5. Provide formal and informal education or training
	5.5. Change practices on multistakeholder governance	→ Intervention 2.2. Frame and reframe the narrative → Intervention 2.3. Rxpose, raise awareness, and denormalise ← Challenge 3. Exploited or misused interventions

Abbreviations: UCI, unhealthy commodity industry; GDP, gross domestic product; ISDS, investor-state dispute settlement; PPPs, public-private partnerships; CoI, conflict of interest.

####  1. Difficult to Achieve or Implement

 Participants noted likely difficulties in achieving or implementing some interventions. Most notably building coalitions and attaining consensus for international treaties, reforming ITIAs and WTO rules, or alternatives to neoliberalism and GDP growth. Participants noted that UCIs would also need to be significantly denormalised through advocacy strategies for there to be sufficient political will to develop national policies, such as reforming political financing systems, and tax laws, due to political opposition. Importantly such policy reforms may themselves be subject to industry sector influence.

 Moreover, many interventions require a significant amount of funding, such as undertaking litigation against UCIs and defending against UCI litigation, and interventions involving the WHO (which has a relatively small budget).

####  2. Partially Formulated or Implemented Interventions

 Interventions need to be comprehensively designed and rigorously implemented or they risk being ineffective. For example, oversight, enforcement and sanction mechanisms are needed to be built into interventions, such as regulating public official interactions with UCIs, lobbying, and revolving doors. Transparency and disclosure mechanisms may also have limited effectiveness if interactions with UCIs are still allowed. Such mechanisms could risk giving a false sense of effectiveness, unless they are paired with sanctions for violations. Participants suggested that interventions need to apply to all UCIs, not a select few, and loopholes need to be closed. Moreover, some forms of strategic litigation may be ineffective since UCI practices are legal. Lastly, adherence to existing interventions, such as Article 5.3. needs strengthening.

####  3. Exploited or Misused Interventions

 Interventions could be exploited or misused. For example, participants warned that changes to investment practices, such as environmental, social, and governance investing, or socially responsible investing would need to be genuine and not co-opted by UCIs as a branding strategy.

 Increasing democratic policy-making is important at all levels of governance, but it may risk enabling third-party groups representing industry to participate in policy discussions. At the same time, advocates should not alienate all industry stakeholders, as they are not always aligned with each other.

 Additionally, UCIs can use some interventions, such as providing their data, to build a facade of good will and distract policy-makers from their harmful practices.

####  4. Interventions Require Tailoring for Context

 Although participants suggested that all of above interventions were needed in most countries, they noted that some specific countries or regions may need specific interventions depending on the UCI strategies to influence policy, and some interventions themselves should be tailored depending on the country or region, or for the particular industry they address. Which interventions were needed or how they should be tailed could be ascertained by conducting a situational analysis. Notably, participants highlighted the significant issue of corruption in some low- and middle-income countries and addressing it would be more appropriate as a first step in tackling UCI influence. Other participants noted that due to political or social culture in less democratic counties, governments may be less willing to implement some interventions, such as regulating lobbying. Therefore the specifics of each interventions may need adapting for what governments or other stakeholders are willing to implement. Participants also noted that interventions needed to be adapted for certain levels of governance (ie, local, national, regional or international levels).

 Moreover, certain UCIs, such as food, may need unique interventions to create fair competition and encourage the production and consumption of healthy alternatives, whilst others may have no healthy alternatives and thus should be ended, such as tobacco.

###  Key Actions to Help Achieve Interventions 

 The following section shows key actions to help achieve interventions (See Table 3 for their interconnectivity and interdependence).

####  1. Coordinate and Cooperate Between Stakeholders

 Participants suggested efforts to create global collaborative networks to coordinate actions addressing UCI practices; for example, between academics, civil society, and policy-makers from different levels of government. These efforts could be facilitated by international governance organisations, such as the WHO.

 Global collaborative networks could involve creating cohesive advocacy coalitions that comprise a wide range of stakeholders. Civil society groups could pool resources together and develop multistakeholder initiatives between governance institutions, academics, and civil society to act as a counterweight to PPPs.

 Networks could advocate for broader political reforms, such as political transparency, democratic governance, and anti-corruption policies and form alliances between governments of low- and middle-income countries to challenge large powerful corporations when developing policy.

 Networks could create international forums to discuss and share knowledge about policy solutions for UCIs between policy-makers from different countries especially including experts in economics and trade.

 Stakeholders should try to find agreement and multilateral support on which industries require regulating. This could be achieved with government support for WHO guidance and advice.

####  2. Invest in Civil Society

 Participants suggested that funding should be provided to civil society and grassroots organisations, such as through earmarked taxes to: expose industry practices; participate in technical policy-making processes; conduct public health campaigns through reframing strategies showing that public health policies will work; create industry watchdogs; and ensure that civil society organisations do not need to rely on industry for funding.

 Participants suggested capacity building for civil society groups on, for example, how to engage in policy consultations and deliberations, and the impact of new technologies, such as digital commerce, on public health.

####  3. Create a Social Movement

 Participants recommended that a social movement should be developed between civil society, activists, and grassroots organisations to address all forms of industry influence on policy, including exposing, denormalising, and showing similarities across, UCIs and their practices. Importantly, this could be achieved through victims of UCIs advocating for effective public health policy.

 Civil society should participate in policy-making processes from local to global levels, and activists should advocate for structural changes, such as alternative forms to economic growth.

 Participants suggested various ways for civil society to be proactive; for example, organise protests; create a complains systems for UCIs marketing; leverage and work with existing fractions within UCIs; develop campaigns on UCI harms using social media; and support political candidates who back democratic reforms and advocate for their election.

 Participants also suggested advocating to gain public support for policy-making process reforms, including democratising policy-making at multiple levels of governance. Additionally, the public could volunteer with organisations that support democratic reforms, meet with local political representatives, and run for office.

####  4. Nurture Leadership

 Participants noted that there is a need for policy leaders or champions in governance institutions to advance and support public health policies and policy-making reforms at national or international levels. This would also help to ensure policy coherence across government departments. However, participants also noted that the existence of policy leaders or champions at the right time is dependent on chance.

 Lastly, participants suggested that leaders who have personal experiences of UCIs’ attempts to influence or deceived them should also speak out against UCIs.

###  Interconnectivity and Interdependence 

**Figure 2:**
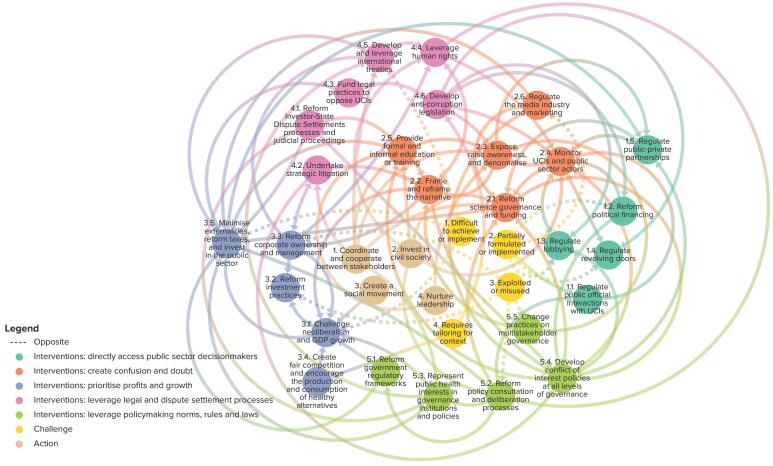
Concept Map of the Interconnectivity and Interdependence Between Interventions, Actions or Challenges. The lines with arrows indicate the that presence of an intervention, action or challenge has an effect on another intervention, action or challenge. The sold line represents a positive direction of travel, ie, the presence of one intervention increased the other, whereas the dotted lines indicate the opposite direction of travel, ie, that would likely hinder the intervention. There are also interconnections that are mutually directed, ie, the arrows going in both directions where the presence of an interventions supports another and vice versa. Abbreviations: UCI, unhealthy commodity industry; GDP, gross domestic product.

 The interconnectivity and interdependence between interventions is indicated by the italicised references above, and shown in Table 3 and Figure 2. For example, adequate enforcement measures, monitoring, and coherent CoI policies across government would be required for lobbying regulations, mandatory disclosure of funding by judicial officials, or research submitted for policy-making consultations. CoI policies would need to detail the consequences for CoI in decision-making, such as removal of public officials from working on policy issues or recusal of judicial officials and arbitrators, which could be overseen by independent governance institution(s). Such an institution could also decide on whether stakeholders actually have CoI in policy-making processes.

 Other interventions would require building public support and changing public opinion to demand governments to act. Exposing, raising awareness, denormalising and changing the narrative by conducting public health education campaigns could be facilitated by UCI and public sector monitoring organisations and social movements. However, such activities and many other interventions entail investing in the public sector and civil society, including legal defence in ISDS processes and judicial proceedings, or capacity building for civil society to engage in deliberative policy-making processes. Funding could come from taxing externalities or a corporate tax baseline, which is unlikely without an international agreement for such taxes, especially within the predominant global neoliberal and GDP growth paradigm. Moreover, without significant policy-making reforms, public participation through deliberative policy-making would be limited.

 Advancing some interventions would be enabled by policy champions, nurtured through training and education about UCI practices, which again would require support and funding.

 Furthermore, including HIAs in ITIAs and national regulatory frameworks would necessitate embedding public health goals in economic costing. And without significant policymaking reforms, public participation through deliberative policymaking would be limited. Lastly, there is a close relationship between developing and leveraging international treaties and reforming ISDS processes; by developing international treaties that protect public health, such as the WHO FCTC, these could be leveraged as counterweights in ISDS arbitration. Additionally, reforms to WTO rules to have stronger public health protections could help to curb UCI ability to leverage these legal processes in their favour. And removing limited liability, leveraging human right through mandatory due diligence, and international human right treaties could improve strategic litigation outcomes.

## Discussion

 This study identified and explored interventions using systems thinking as a theoretical lens to develop a broad list of interventions and encourage the consideration of system complexities when addressing UCI influence on public health policy. Workshops allowed for participants to critically engage with how interventions related to the systems map. Participants identified 27 disparate and heterogenous – yet interconnected and interdependent – interventions aiming to prevent and mitigate UCIs’ ability to influence different parts of the system. Participants also suggested four key challenges and actions to help achieve interventions.

 This study brings together and consolidates these proposed interventions coherently for the public, policy-makers and public health practitioners, who may not be familiar with them. This study draws together three distinct ways the literature frames interventions around UCI influence, thus showing their interconnectivity and interdependence: (1) interventions as governance mechanisms in policy-making processes, (2) interventions aimed at structural factors that give power to UCIs, and (3) interventions as actions for academics and civil society to counteract UCI practices.

 Firstly, the types of interventions most consistent with public health literature are governance mechanisms by Mialon et al^18^ which correspond largely to the systems map pathways: *directly accessing public sector decision-makers*; *creating confusion and doubt about policy decisions*; and *leveraging policy-making norms, rules, and processes *(See Table 1). As mentioned above, these governance mechanisms aim to manage, increase transparency, or prohibit CoI and interactions with UCIs, or identify, monitor and educate stakeholders about UCI practices and harms. While these interventions are important, they mainly apply to policy-makers and researchers, and do not seem to acknowledge the other parts of the system or underlying structures that may limit the effectiveness of such interventions. Other public health scholars have similarly suggested such interventions for these pathways, namely teaching corporations as structural causes of disease in public health curricula^27^; prohibiting UCIs donations to international governance institutions^60^; using HIA in ITIA negotiations^61^; researchers refusing industry funds^27,62,63^; earmarking tax from industry to fund research for which researchers could competitively apply^62^; regulating election campaign donations,^18,23,64^ and independently monitoring or managing PPPs.^65,66^ Interventions need to acknowledge the limitations of interventions that mostly focus on UCI ability to influence this part of the system.

 Secondly, interventions identified in this study corresponding to the pathway *prioritising corporate profits and growth *are consistent with interventions to address structural factors that give rise to UCIs’ power and enable their use of political practices.^24^ These intervention include, for example, degrowth,^67-69^ tax and subsidies reform,^22,23,35,70^ alternative investment practices and business models,^35^ including removing corporate right to personhood or limited liability.^26^ Public health researchers have also suggested working with trade and supply chain stakeholders to help achieve public health goals.^28,31,71,72^ Interventions that address this pathway resemble solutions to CDoH more generally and are not obviously applicable to the policy-making process.^35^ Although the interventions herein are targeted more towards political practices, others are relevant to targeting other corporate strategies ie, science, marketing, supply chain, labour and employment, financial and reputation management practices.^35^

 Thirdly, literature discusses strategies that actors could engage in to counteract industry influence. For example, “link[ing] with and learn[ing] from social movements to foster collective solidarity,”^21^ gaining political commitment through coordinated advocacy networks,^73,74^ building coalitions, and undertaking advocacy campaigns,^35^ and exploiting division within and across industries.^70^ These correspond to actions to help achieve interventions and to the pathway *leveraging legal and dispute settlement processes. *Researchers suggest strategic litigation as an important lever for change^75-79^ that pressures governments to develop both effective health policy *and* the above interventions to address UCIs influence. Strategic litigation involves the use of ethical arguments^35^ that align with leveraging human rights and framing and reframing the narrative to help build support for a social movement.^80,81^ Recent research into the right to enjoy the benefits of scientific progress could help advance public health goals, but which may be inhibited if vested interests are prioritised.^82^ Importantly, scholars note that social mobilisation^21,35^ is crucial to achieve systems change.^83,84^

 Strategies to counteract industry influence align with “healthy alliances,” defined as “a partnership for health gain that goes beyond healthcare and attempts collectively to change the social and environmental circumstances that effect health.”^85^ Healthy alliances are underpinned by the idea that collaborative efforts between organisations rely on the same essential factors crucial for any successful alliance to thrive.^85^ These factors include a shared purpose, a level of mutual trust, and adequate benefits for participants to work together.^85,86^ According to the literature, a helpful way to achieve successful healthy alliances is understanding the interpersonal and social processes involved.^86^ To implement actions to help achieve interventions, public health actors could look to apply healthy alliances frameworks particularly at multiple levels of governance, in order to achieve collaboration, coordination and a shared purpose for working towards these interventions.

 Using a systems thinking lens, the above research shows a wide range of disparate and heterogenous interventions, but to date has lacked a coherent unifying framework to indicate their interconnectivity and interdependence. This study also reiterates the importance of many interventions to stakeholders; although many may not be “novel” and have been previously proposed, some are absent in the most recent literature on ways to address UCI influence on public health policy, such as human rights approaches, reforming policy consultation and deliberation processes, reforming ISDS processes and judicial proceedings, industry monitoring, investing in civil society, nurturing leadership, and reliable funding for interventions. It is therefore important that they are presented here as key options going forward.

 Systems thinking scholars suggest that interventions with highest potential impact and most feasible should be prioritised.^53,57,87,88^ Changing entrenched system structural factors^52,53,89^ – that are sources of UCI power, for example, neoliberalism and GDP growth – may be most impactful, but immensely challenging to achieve.^90^ Changes to this require global agreement and cooperation between governments on global economic, financial, and banking systems^1^ and also buy-in from people across the world to practically change their consumptogenic lifestyles^91^ to that of sustainable consumption and “living locally.”^92,93^ It is likely that, for the present, people may want to retain their lifestyles. Politicians are also not incentivised to develop degrowth policies due to constituents largely not voting for political parties who are sympathetic to these policies, though this may be changing.^94^ Theorists have argued that even if politicians who promise to develop degrowth policies were elected, these policies will not work unless there is broader global transition to accepting “degrowth-oriented” values.^90^

 Arguably, more feasible yet less impactful interventions may tackle “visible symptoms”^53,57,87,88^ of industry influence in the system aimed at reforming policy-making processes. Again, this would require pressure from citizens through social mobilisation or elections, for example. But even if policy-making processes are sufficiently accountable, transparent, democratic or participatory, the assumption is that sources of power, if not addressed, would still bear weight on these processes; there would be a constant risk of such processes being undermined in favour of industry preferences. In other words, taking a piecemeal approach and overlooking the underlying causes, or only seeking to change some parts of the system but not others, could allow UCIs to adapt to interventions and find alternative pathways to influence policy.^48,53,54^ As such, interventions to address systems change are likely to require a holistic and coherent approach to be effective.^37,41,42,48,53,95^

 Although this paper does not evaluate effectiveness of these interventions, growing literature shows that systems thinking can do so.^96^ Using empirical data, for example, surveys or questionnaires, informal qualitative feedback, health measures, and population health or education datasets,^97^ research suggests that systems interventions have the potential for addressing complex problems.^96-99^ In the absence of empirical data, future research could develop system dynamic models to simulate the interactions within the system to explore the effects of potential interventions.^99^

 Furthermore, a growing volume of literature support the need for intervention synchronicity.^50,98,100-104^ For example, to address social determinants of health in local urban spaces, stakeholders have needed to work across healthcare, social care, housing, education, transport, environmental sectors and have also engaged with voluntary community and social enterprises.^50,105,106^

###  Strengths and Limitations

 There are several strengths and limitations to this study. Firstly, although a wide range of participants contributed, interventions herein are not likely to be exhaustive, and some may not have proven effective or may not have been implemented. A participatory systems mapping lens can promote creative, aspirational and theoretical ways to achieve change, and in this case to suggest potential interventions, but further research using empirical methods should determine intervention effectiveness and feasibility.

 Secondly, workshops could have benefited from in-person and prolonged participant engagement, which would have facilitated richer discussions. Although this was not possible due to the COVID-19 pandemic, a strength of this study was the online format which allowed for engagement with a diverse range of stakeholders from different geographical regions, across the world who otherwise would not have been able to participate. Workshops also allowed for participants to critically engaged with the systems map even if some components were missing. Notably, participants with expertise in other UCIs, such as fossil fuels, firearms, social media, and gambling, were not included. This may have impacted the suggested interventions. Further research could engage participants with expertise in other UCIs to explore what interventions could apply and how.

 Thirdly, in analysing the data, it was not always clear to which part of the system an intervention applied. Indeed, the same interventions may apply to different parts of the system simultaneously and, depending on how interventions are framed, they could be considered analogous.

 Lastly, participants made broad suggestions and found it difficult to elaborate on what interventions entailed. Participants suggested broad intervention “principles,” such as increasing transparency, accountability or state power, or reducing industry power. For other interventions, such as lobbying and revolving door regulation, or CoI policy, participants had difficulty describing what these would look like, and how they would be implemented, and what level of impact could have on the system. Perhaps this could be due to the fact that some of these interventions are poorly defined and understood, in particular, those concerning CoI.^107-109^ Similarly, participants found it challenging to determine the likely effectiveness, or relative impact, and feasibility of interventions during workshops given the complexity of the system, which highlights the need for further in-depth discussions and research into this aspect of interventions.

###  Further Research

 Given these limitations, there are opportunities for further research. Firstly, research should continue to identify interventions – whether theoretically or empirically – and clarify what they might entail and their feasibility, especially in different context, and whether they apply to other UCIs, such as fossil fuels, firearms and gambling industries.

 Secondly, this area lacks clear language with which to describe the complexity of interventions and how they interlink. Research could therefore develop a nomenclature or heuristic to help stakeholders think critically about how their own interventions interact with others and work within the system.

 Thirdly, there is a need to have an empirical approach to evaluate the effectiveness of interventions and their interconnectivity and interdependence. However, such research may be challenging due to the complexity, and limited instances of partly or fully implemented interventions. For interventions that have been implemented, data may not be easily available.^110^ If there is a lack of data, however, logic frames^111-113^ or theories of change^114,115^ could help design, implement and gauge likely intervention effectiveness, and other specialised methods could measure the robustness of particular interventions, such as lobbying laws.^116^

## Conclusion

 Applying systems thinking as a theoretical lens revealed that interventions to UCI influence on public health policy should be considered holistically, taking into respect diverse parts of the system and how they interact. Interdependence and interconnectivity between interventions suggest that they need to align and work together to be effective. Policy-makers, civil society, academics and media all need to recognise and acknowledge this complexity and interdependence. Only then can they best collaborate to coordinate and package interventions into the most effective prevention and countering strategies.

## Acknowledgements

 The authors would like to thank all workshop participants for giving their time and sharing their invaluable expertise. Fully acknowledged participants are placed in alphabetical order: *Alice Fabbri*, Tobacco Control Research Group, University of Bath; *Andrew Rowell*, Tobacco Control Research Group; *Angela Carriedo*, World Public Health Nutrition Association; *Belinda Townsend*, Australian National University; *Ben Hawkins*, MRC Epidemiology Unit, University of Cambridge; *Britta Katharina Matthes*, Tobacco Control Research Group, University of Bath; *Caroline Cerny*, Obesity Health Alliance; *Charles DH Parry*, Alcohol, Tobacco & Other Drug Research Unit, South African Medical Research Council; *Crispin Acton*, former civil servant, United Kingdom; *Daniel Dorado*, Corporate Accountability; *Debby Sy*, Global Centre for Good Governance in Tobacco Control, a STOP Partner; *Dhamaravelli ( Vimla ) Moodley*, former civil servant South Africa; *Fran Baum*, Stretton Health Equity, University of Adelaide; *Hazel Cheeseman,* Deputy Chief Executive, ASH; *Jaime Arcila*, Corporate Accountability; *Jane Martin,* Obesity Policy Coalition; *Jennifer Lacy-Nichols*, University of Melbourne; *Kate Oldridge Turner*, World Cancer Research Fund International; *Kathrin Lauber*, University of Edinburgh; *Kathryn Reilly*, former member of Irish Parliament, *Leigh Haynes*, Framework Convention on Global Health Alliance; *Leslie London*, School of Public Health and Family Medicine, University of Cape Town; *Lilia Olefir*, NGO Advocacy Centre “LIFE”; *Lori Lake*, The Children’s Institute; *Lucy Westerman,* VicHealth; *Maik Dünnbier*, Movendi International; *Marian Lorena Ibarra*, Food Policy Program, Global Health Advocacy Incubator; *Mark Tomlinson*, Institute for Life Course Health Research, Department of Global Health, Stellenbosch University; *Martin McKee*, Professor of European Public Health, LSHTM; *Mateusz Zatoński*, Tobacco Control Research Group, University of Bath; *Mike Daube*, Curtin University, Western Australia; *Mindaugas Štelemėkas*, Head of Health Research Institute, Faculty of Public Health, Lithuanian University of Health Science; *Nason Maani*, Global Health Policy Unit, University of Edinburgh; *Nicholas Freudenberg*, CUNY School of Public Health; *Nina Renshaw*, NCD Alliance; *Øystein Bakke*, Global Alcohol Policy Alliance; *Patricia Lambert*, International Legal Consortium at the Campaign for Tobacco-Free Kids; *Paula Johns*, ACT Health Promotion, Brazil; *Pepita Barlow*, Department of Health Policy, LSE; *Peter Rice*, European Alcohol Policy Alliance; *Petronell Kruger*, The SAMRC/Wits Centre for Health Economics and Decision Science (PRICELESS SA); *Raquel Burgess*, Department of Social & Behavioural Sciences, Yale School of Public Health; *Rima Nakkash*, George Mason University; *Sarah Dance*, Tobacco Control Research Group, University of Bath; *Sebastian Rositano*, Commercial and Economic Determinants of Health Unit, WHO; *Sharon Friel*, Australian National University; *Sulakshana Nandi*, Public Health Resource Network Chhattisgarh India; *Susan Goldstein*, SAMRC Centre for Health Economics and Decision Science PRICELESS, School of Public Health University of the Witwatersrand; *Tara Van Ho*, University of Essex; *William F Lamb*, Mercator Research Institute on Global Commons and Climate Change (MCC). The following participant prefer to be acknowledged by only their affiliation: Oregon State University. One participant preferred not to be acknowledged.

 Authors would also like to acknowledge Melissa Mialon and Mark Petticrew for early discussions prior to the development of the study, and Selda Ulucanlar, Bryan Clift and Harry Rutter for their input on analytical methods.

## Ethical issues

 Ethical approval (EP20/21002) was granted by Research Ethics Approval Committee for Health (REACH), University of Bath. Informed consent was obtained from participants, and all were given the option of being openly acknowledged for participating in the workshops.

## Competing interests

 The authors declare that they have no competing interests, financial or otherwise, related to the current work. To the best of our ability, this also includes the late Mateusz Zatoński.

## Funding

 AB received a PhD studentship from University of Bath. AG is supported by the UK Prevention Research Partnership (MR/S037519/1), which is funded by the British Heart Foundation, Cancer Research UK, Chief Scientist Office of the Scottish Government Health and Social Care Directorates, Engineering and Physical Sciences Research Council, Economic and Social Research Council, Health and Social Care Research and Development Division (Welsh Government), Medical Research Council, National Institute for Health Research, Natural Environment Research Council, Public Health Agency (Northern Ireland), The Health Foundation and Wellcome. KB is supported by the UK Prevention Research Partnership (MR/S037586/1). AVDA is funded through PhD funding from the University of Bath, in affiliation with the SPECTRUM consortium (MR/S037519/1). SPECTRUM is funded by the UK Prevention Research Partnership (UKPRP). MB, BKM, SD, and MZ are or were funded through Bloomberg Philanthropies’ Stopping Tobacco Organizations and Products funding (https://www.bloomberg.org/). None of the funders had any role in the study design, data collection and analysis, decision to publish or preparation of the manuscript.

## Supplementary files

Supplementary file 1. Participants’ Suggestions and Illustrative Quotes.

## Additional Information

 Our dear co-author Mateusz Zatoński, PhD, sadly died on January 17, 2022.
