# Supplementary material for: Complex Interventions for a Complex System? Using Systems Thinking to Explore Ways to Address Unhealthy Commodity Industry Influence on Public Health Policy
Source: Int J Health Policy Manag. 2024 Apr 7;13:8033. doi: 10.34172/ijhpm.2024.8033 (PMC11608292; doi:10.34172/ijhpm.2024.8033)
Supplement: Supplementary file 1 — Participants’ Suggestions and Illustrative Quotes. [file ijhpm-13-8033-s001.pdf]

Article title: Complex Interventions for a Complex System? Using Systems Thinking to Explore Ways to Address Unhealthy Commodity Industry Influence on Public Health Policy

Journal name: International Journal of Health Policy and Management (IJHPM)

Authors' information: Adam Bertscher[1]*, Britta Katharina Matthes[2], James Nobles[3], Anna B Gilmore[2], Krista Bondy[4], Amber van den Akker[2], Sarah Dance[5], Michael Bloomfield[1], Mateusz Zatoński[2]

[1]Department of Social and Policy Sciences, Faculty of Humanities & Social Sciences, University of Bath, Bath, UK.

[2]Department of Health, Faculty of Humanities & Social Sciences, University of Bath, Bath, UK.

[3]Centre of Active Lifestyles, Leeds Beckett University, Leeds, UK.

[4]School of Management, Marketing, Business & Society, University of Bath, Bath, UK.

[5]Department of Psychology, Faculty of Humanities & Social Sciences, University of Bath, Bath, UK.

Additional Information: Our dear co-author Mateusz Zatoński, PhD, sadly died on January 17, 2022.

***Correspondence to:** Adam Bertscher; Email: ab3298@bath.ac.uk

Citation: Bertscher A, Matthes BK, Nobles J, et al. Complex interventions for a complex system? Using systems thinking to explore ways to address unhealthy commodity industry influence on public health policy. Int J Health Policy Manag. 2024;13:8033. doi:10.34172/ijhpm.2024.8033

Supplementary file 1. Participants' Suggestions and Illustrative Quotes

Table S1. Interventions to reduce UCI ability to directly access public sector decisionmakers

Table S2. Interventions to reduce UCI ability to create confusion and doubt about policy decisions

Table S3. Interventions to reduce UCI ability to prioritise their growth and profits

Table S4. Interventions to reduce UCI ability to leverage legal and dispute settlement processes

Table S5. Interventions to reduce UCI ability to leverage policymaking norms, rules, and processes in their favour

Table S6. Potential challenges to advance interventions

Table S7. Key actions to help achieve interventions

Please note in following tables the illustrative quotes are taken from workshop transcripts and written whiteboard (sticky note) comments, the former of which is indicated with ‘SN’.

Participant references are as follows: academia (A), civil society (CS), former public offices (FPO), and global governance organisations (GGO)

Table S1. Interventions to reduce UCI ability to directly access public sector decisionmakers

Intervention category	Intervention description	Illustrative quotes
**1.1. Regulate public official interactions with UCIs**	Governments should develop clearly defined mandatory rules of engagement or codes of conduct for public officials by detailing how they should be permitted to interact with representatives of UCIs (A14, CS12, A6, CS9, FPO3, CS15, CS16, CS17). There should be enforcement and sanctions for violations of such rules or codes (A14, A19, A16, CS15) and improved enforcement and implementation of WHO FCTC Article 5.3. (CS17, A26, CS18), for representatives of all government departments, not just departments for health (CS20).	*“…with direct access to policymakers… whilst we have Article 5.3, one of the biggest concerns is that we don't have a code of conduct for policy, so as a result of that… direct access to policymakers is an open door, so industry uses every opportunity…”* (FPO3)<br><br>*“…They did have ways to be inside* [policymaking discussions] *through others* [people/organisations]*… So, they were not, of course, completely outside, but formal was outside… maybe the informal role they had where they were still able to do it* [UCIs to lobby policymaker], *but… formally they were outside.”* (A6)<br><br>*“…there are challenges around rules of engagement…they can be weakened in themselves or not adhered to, and the standards are really low. So, I think I would definitely put some definition and robustness behind rules of engagement, 'cause I wouldn’t assume that they're good just because you have them.”* (CS15)<br><br>*“…if you go and spoke to our Ministry of Commerce or a finance ministry and the first thing that they mentioned is that the Framework Convention of Tobacco Control do not (sic) obligate or do not generate any obligations for them - that this is something that only applies the ministries of health…. So, I feel that out of this systems mapping if authorities or these policymakers understand this conception, this will also allow them to understand that the industry or corporations interfere in different layers and that do not only target the ministries of health and open this dialogue.”* (CS20)
**1.2. Reform political finance**	There should be enforced restrictions on political donations to politicians, political parties, or election campaigns (FPO4, A4, CS18, A16, A25, A24, CS9, A16), and real-time reporting and	*“…not allowing money to enter politics in any meaningful way, either through the direct funding of political parties or individuals or election campaigns, or other modes like this… you have to declare donations.”* (A4)<br><br>*“…real-time reporting of all political donations would be really important. That's a big way that they* [UCIs] *get influence…”* (A10)

	disclosure of all such political financing (A10, A4, CS9).	*"…in our case here, where we did have, it was allowed* (sic) *to fund their* [politicians'] *campaigns by corporations, were allowed to fund campaigns… and then it was forbidden. But what happened now, they still fund, and we don't have information about that…"* (CS9)
**1.3. Regulate lobbying**	There should be a complete prohibition of UCIs from lobbying (A20, CS11), by developing publicly accessible lobbying registers (A25, A5, A23, FPO4, FPO2, CS4, A24), consisting of mandatory disclosures, including of gifts CS18). This should involve monitoring and the timely reporting of all lobbying practices directed at public officials (A5, A23, A25, A17, CS11, A4, FPO2, CS18, CS3, CS2, CS8, CS20), including judicial officials (A25). This type of regulation should be balanced with the need of policymakers to carry out their duties and interact with the necessary stakeholders (A23). Importantly, it is essential to have adequate oversight and enforcement procedures for lobbying registers, (FPO2, A16, CS8, A25), and mandatory disclosures of lobbyists' CoI (CS11, CS18, FPO4, A25).	"*I found that in our own experiences departments haven't been recording the meetings, so when you do maybe find a reference to a meeting that took place or a communication, there's nothing there. No department has it, so you're missing out on vast swathes of information around the policy may have come about..."* (FPO2)<br><br>*"…lobbying regulation necessities and the clear transparent regulation on the lobbying activities how we define in every country is very important."* (A5)<br><br>"*…how do you formulate a* [lobbying] *policy that kind of captures the stuff that you want to capture but doesn't completely hamstring government, doesn't mean that people* [policymakers] *can't do meetings* [with any non-government actors]?<br><br>*"Mandatory disclosure of lobbying and gifts from companies/lobbying companies to politicians. Mandatory lobbying register of meetings between governments and lobbyists whether direct or third party; Mandatory register of interests/gifts of politicians and senior civil servants."* (CS18-SN)
**1.4. Regulate revolving doors**	Revolving doors practices should be prohibited (A25, CS3, C17, CS4, A12, CS15) by restricting the number of years before public officials can work for the private sector, or vice versa (CS1, CS10, A15, FPO2), and prohibit public sector officials from having second jobs (A4). There needs to be oversight and enforcement of revolving doors regulation (A25, A16, FPO2) and removal of public officials implicated in such practices (A25).	*"…there are lobbying registers, but it does need to make sure that… there're no loopholes, that there are the cooling off period* (sic)*… and enforcement mechanisms, that there are the prosecutions, that you can't just go over* [the cooling off periods]*…"* (FPO2)<br><br>*"And if there's somebody really monitoring that* [revolving doors], *and if there's enforcement of the regulations themselves…. It's like the people who are supposed to be responsible for tabulating and all this data and reporting is coming in and like have a small base basement office…. There's the need for improved regulation of activities and of reporting, and then there's dissemination of information and the enforcement of the regulations. So that's on lobbying the same kind of thing is applicable to, of the revolving and reverse revolving door* (sic). *People coming in from industry, there needs to be stricter regulations and their needs to be some people saying about who can come into government from industry, but while they're in office, regulations about what… policy decisions, and in law, regulations that they can be involved in and when they should be required to recuse themselves* (sic) *and increased declarations of their conflict of interest"* (A25)

		"…it's really also the culture of transparency and integrity and… anti-corruption so that sort of needs to play a key role in here…that should respond to the issues that were raised… around transparency… industry tactics, revolving doors, that everything in there has to do with… policies involving governance." (CS17)<br><br>*"…you see prominent politicians being part of the executive boards of big companies…. It's kind of a revolving door problem, but it's also… giving them direct vested interest in how these companies perform…"* (CS4)
**1.5. Regulate public-private partnerships**	Standards for managing or forming public-private partnerships (PPPs) at all levels of governance should be developed (GG01, CS8), including financial transparency (CS3), and monitoring PPPs (A21, CS3). There should be mandatory disclosure of CoI for those involved in PPPs (CS11, CS6, CS20, A7), and prohibition on involvement in PPPs if CoI are found (CS11, A15). Moreover, independent evaluations of PPPs should be conducted (A18, A23).	*"Corporate capture, at the global level with multilateral bodies, for example, is what they love to position, which is multistakeholderism. And that's why it is difficult to go to WHO, PAHO [Pan American Health Organization] or any other of these multilateral bodies when they are already co-opted by the industry. So, what the industry is doing is they are looking into those entities at the global level, that set the rules or the guidelines for policy development at the country level and those bodies are already co-opted by the industry. So, trying to think of strategy* (sic) *from the advocacy perspective to do something. Or maybe thinking about having governments or that level of decision-making in policy guidelines…"* (CS8)<br><br>*"Regulatory measures to improve financial transparency of PPPs."* (CS3-SN)<br><br>*"…authorities' to implement mandatory codes of conduct to disclose conflicts of interest, to disclose that in more detail the public-private partnerships that exist. When you talk about public-private partnerships… but if you start digging there is a lot of things and… usually there is no disclosure of any or no conflict of interest* [policy]*."* (CS20)<br><br>*"…what we thought we had was a government cabinet memo saying there should be no direct working with the liquor industry partnerships with the liquor industry, but it's got* (sic) *eroded and no one can find it anymore. But that sort of thing* [government memo] *has quite a lot of power… because it's an intervention which can be used to block the industry in multiple ways."* (A15)<br><br>*"Independent evaluation of PPPs"* (A18-SN)<br><br>"*Independently commissioned evaluations* [for PPPs]*"* (A23-SN)

Table S2. Interventions to reduce UCI ability to create confusion and doubt about policy decisions

Intervention category	Key intervention details	Illustrative quotes
**2.1. Reform science governance and funding**	Governments should establish independent public oversight bodies, such as public health agencies, to monitor and evaluate public health evidence to a benchmark standard for policymaking purposes (FPO4, A14,	*"… one element of the solution to resist industry's policy influence is to have independence in the policymaking process, is at least you can have an independent body that provides evidence and maybe gives advice… that was part of the intention in creating Public Health England, although it was never technically independent, there was some intentions to treat it as independent…"* (FPO4)

	A13, A15, CS5, CS4, CS3, A24). Such governing institutions could decide on consequences for the presence of CoI in research submitted for policymaking purposes (CS12, A15) and should monitor the progress and goals for regulating unhealthy commodities, (A14), whilst being reliably and adequately funded (CS4, CS3, CS10). Participants also suggested that civil society or independent academics should be required in the oversight of industry research compliance. (CS10).<br><br>There should be mandatory disclosure of CoI in private sector research submitted for policymaking purposes (A15), including disclosure of funding of researchers and think tanks (CS16). Universities, professional associations and academic journals should develop CoI policies (A13, CS12). Alternative funding systems and rules for research on unhealthy commodities should be established that reduces UCI ability to influence research (A17), such as adequate public funding of science on unhealthy commodities (A13) or restricting UCIs from funding academics (CS4).	*"…[an] independent foundation that looks at the corporate and social and economic determinants of health and really tries to address it with substantial fundings."* (A14)<br><br>*"…govt departments to link who can do what in a professional administrative process to a benchmarked standard* (sic) *- that would close the door to industry's back door on research and researchers who are not qualified."* (A14-SN)<br><br>"…*funding for public health interests* [by government] … *like the public health institutes... the Thai health model… certain percentage of the levies on alcohol and tobacco is put back into public health work by the Thai Health Promotion Foundation in Thailand…"* (CS4)<br><br>*"Firstly, there are rules about what evidence gets onto a policy agenda and how. If government takes anything, then there has to be an active and informed process of filtering and valuing evidence; secondly, there need also be rules about firewalls, some countries and agencies do have such rules, others don't. Then the public consultation rules - in general, though not always sufficient, transparency operates in the direction of fair decisions. Industry influence behind closed doors is far worse… [than] industry influence exposed to the public glare. Then all the ways in which evidence is weighed… or similar kind of evidence-weighing approaches* (sic)*."* (A15-SN)<br><br>"*Incorporation of considerations of Conflict of Interest into professional performance evaluation of public services – there should be consequences*" (A15-SN)<br><br>"*It's not just about declaring the conflict of interest. There's a question around what happens next. And how do you ensure that that conflict of interest is not damaging to the integrity of the research to public health, to child health, whatever the overarching primary purpose is.*" (CS12)<br><br>*"…whatever we* [are] *looking for in the regulations… making sure that there are things like the test cycles that are underpinning it* [evidence], *to stack up…. you've got to have kind of civil society or kind of coupled, maybe with independent academia that can make sure that…implementation and enforcement is… what it should be."* (CS10)<br><br>*"…this is also about science…there is an obligation on states to ensure that people benefit and enjoy the benefits of science, scientific progress as it's framed, so… the question of funding, the health promotion programs or research into health promotion should also be a state obligation. You know whether they take the money from tax or some other sources or how they organise it that it has to be quality science. So, I think there's sort of opportunities to leverage that as well….* (A13)<br><br>"*Clear restrictive rules: no support from tobacco or alcohol industry is allowed for academia etc. Implemented by Government regulation* (sic)*"* (CS4-SN)<br><br>"*Ensure conflict of interest policies in research organisations and institutes of higher learning…*" (A13-SN)

**2.2. Frame and reframe the narrative**	Participants suggested numerous ways to frame and reframe narratives around unhealthy commodities and UCI influence on policy, for example, framing public health issues as a matter of social justice (CS15, CS6, CS5, A10), that those suffering from NCDs are victims of UCI practices (CS15, CS5, A10, CS17), and that UCIs make profit from death (A7). Participants suggested framing UCI influence on policy as a matter of corruption (GG01, A9, A11), and social justice and violating democratic principles (A9); and framing unhealthy commodity consumption as socially unacceptable (CS5), part of the climate change agenda (CS15, CS1), and not as the result of individuals' choice or responsibility (CS1, CS15, CS16, CS5), but due to UCI influence on individuals' behaviours (CS15, CS6, CS12, A24).<br><br>Participants also suggested framing that corporations should be accountable for their actions analogous to natural persons (A23); that UCIs have a CoI in public health policy (CS6); UCI regulation would create a healthier society providing economic and business benefits (CS15, A22); it is inappropriate for UCIs to make scientific claims about the medical or public health impacts of their products (A23); and that UCIs do not have moral autonomy or authority (A24).<br><br>Participants suggested challenging the frame that UCIs are 'good citizens' or benevolent actors (A1, A3) and industry	*"… [there is a] lack of a sense of victimhood as a result of kind of some of the wrongs by corporations in some of these other spaces"* (A10).<br><br>*"…if you couldn't articulate your policy ask* (sic) *within the realm of climate change, then you're not relevant…"* (CS15)<br><br>*"But even in high income countries you can show that better health is associated with greater productivity. Greater labour force participation. Greater investment in your family and your education and […] your business and so on and so forth. So, therefore, that would argue that corporate behaviour that damages health is actually inimical to economic growth. So, the argument that the regulations you're putting in place to constrain the activities of corporations is undermining economic growth is just nonsense."* (A22)<br><br>*"…in a fully-fledged fair democracy when someone with considerably greater resources than you have such influence over policy… there's an injustice in kind of democratic processes* (sic)*."* (A9)<br><br>*"…the concept of tobacco victims and involving them in advocacy campaigns and their voice as important is heard, is one of the classical interventions. But what we see in reality, what I've seen for practice from my actual work, that it's very hard to involve those people who actually suffer from the industry."* (CS5)<br><br>*"Well, we do know that they control the narrative in the media and the cultural perception of smoking and that's* (sic) *it's your personal choice and how they created this image that, at the end, people, many people who do suffer and then their relatives – they don't feel like they have a right to ask for justice…"* (CS5)<br><br>*"…communities will be hit disproportionately hard by climate change. So, I think there is an equity issue that's really important here… Which goes back to your framing about the social justice thing. But that's most probably slightly more moderate language than the kind of victim type thing, which I don't know if we play into that kind of stigma issue..."* (CS15)<br><br>"*And rather than sort of talking about things* [public health regulations] *punitively, you could talk about them positively, like healthy environments, like positive actions to do that…thinking about framing how things don't always have to be polar opposites. They* [public health regulations] *can be positive for business but positive for health as well and looking for those opportunities for that, the kind of better spaces to occupy together."* (CS15)<br><br>"*…stigma is the first thing that comes to my mind, certainly in food that people with… obesity are so stigmatised that I'm not sure how useful it is while that stigma is still so prevailing."* (CS16)

	self-regulation and PPPs are altruistic initiatives (FPO4). Lastly, participants suggested targeted framing or reframing the narrative at decision-makers who have vested interests in UCIs (CS2, A6, CS14) or who support neoliberal policies (CS14).	
**2.3. Expose, raise awareness, and denormalise**	Participants suggested that UCIs practices should be exposed to help raise awareness, and thus denormalise unhealthy commodities and UCIs (A25, A19, CS4, A26, FPO2).<br><br>Firstly, participants suggested exposing: the relationships between public officials, heads of UCIs, and UCIs association representatives (A26, CS5); overlaps of boards of directors between UCIs (A13); issues with multistakeholderism and PPPs (CS3, GG01, A3) and corporate social responsibility (CS1, A19, CS3). Moreover, exposing how UCIs influence evidence (CS3); harms caused by UCIs products (A1), consumer misinformation (CS1), the use of UCI front groups and funded organisations (A26), and issues of poor transparency in policymaking (A1).<br><br>Secondly, one could raise awareness of UCI arguments to 'inoculate' or prepare the public for them (A20, A4, A1, CS6) and to foster public outrage about issues of UCI influence (FPO2) to help change public opinion and demand government to action (CS13, GG02, CS5, A18, CS3).	*"The more they're exposed, shine a light on them, the more difficult it is for them to pursue those approaches…. expose industry, expose governments where they're doing* (sic), *we're seeing inappropriate things are being done. Expose the funded groups. Expose the supporters."* (A26)<br><br>*"…it's a catch 22. In order to expose the problems or in order to try and shine the lights on each change you need to expose the problems."* (FPO2)<br><br>*"…in food we need very big work in evidence: who is doing the evidence? Who is sponsoring the evidence? What is being said by the industry? Because obviously* [for] *tobacco we know it's a product that is not… good for you…"* (CS3)<br><br>"*I've spoken a lot about transparency because this sort of idea of exposing all these companies are* (sic) *involved in corporate social responsibility and it's just such a great protection. It's their armour in some respects and dismantling that I think can be very powerful as a… strategy…*" (CS1).<br><br>"*But a lot of the time that comes down to ourselves… to researchers to try… make a bit of outrage about it* [UCI influence], *but unfortunately just doesn't really exist… And while it might exist after… typical opposition level, or it might exist at NGO researcher, academic level, it doesn't exist with… the ordinary public. And unless… there's more of an awareness around these issues with ordinary people, they'll never be that kind of public and political appetite to take it on."* (FPO2)<br><br>*"…there's a need for ongoing awareness. There's a need for ongoing orientation to ensure that politicians, decisionmakers, private sector individuals, civil society are actually aware of the need to want to do it or to want effective interventions…"* (FPO3)<br><br>*"…* [academics and NGOs should] *work with the media and general society, basically to educate them on not why the specific products are bad, but why the specific measures are introduced. There are very good explanatory videos on the internet, but they are not provided to the general public, and it's related with… a specific projects* (sic) *that needed to dedicate their attention to this."* (A5)<br><br>*"…some work we're doing on food, we're trying to denormalise in a similar way, predatory marketing of unhealthy food to vulnerable populations to make it to… large sections of the population, elected officials, the public, maybe even some businesses* [to] *say that's wrong…"* (A8)

	Thirdly, participants suggested denormalising UCI products and practices by exposing UCI practices (A26, A8, CS6, CS8, CS9), as suggested above, by: working with journalists (CS14, A6) and using online and offline media, including social media (CS5, CS2, CS1, A5, FPO3, A15, A25); reporting on decisionmakers' interactions with UCIs, their CoI, and corrupt practices (CS5); using public relations strategies, such as leveraging medical professionals and experts to convey public health messages (CS14); sharing personal experiences of UCIs attempts to influence policy (A6, CS5); translating academic research into communication products that resonate with policymakers and consumers (CS12); and targeting decisionmakers by communicating the impact of UCIs on health (A1, FPO3, CS2).	*"…delegitimation of what the industry is doing, and that's a very important point. If you look across commodities* [UCI] *interests, their main interest is to maintain the brand, maintain the corporations' relationship with consumers, I think that's the most important part."* (CS8)
**2.4. Monitor UCIs and the public sector actors**	Participants suggested monitoring UCIs by establishing civil society watchdog groups to: political practices (A15, CS3, A14); influence on science (CS18, CS3); financing activities (CS3); mergers and acquisitions (CS3), global corporate tax payments (A25); and violations of, and inappropriate, marketing practices (CS12, A21, A24). Stop Tobacco Organization and Products (STOP) was noted as good example of industry monitoring (A26). There should also be monitoring of decisionmakers' voting behaviours in parliament (CS3), and legislation should be developed to institutionalise	*"…I think this is a very important thing to question who is doing the science… I agree with NGOs watchdogs, but also to track… mergers and acquisition of big corporations"* (CS3).<br>*"…what you've got in the tobacco area… the monitoring of industry tactics…. We're quite disorganised in alcohol field, but we need not only a framework convention, but we need the people, there are good people… we could do better if we had some more resources to expose to put pressure on government to be in the media… there's no magic bullets… you've had a number of forums which do it, and I think they click, they're quite effective, but they got to be sustained. And what happens when we retire? And who's going to take over and keep all these things going?"* (A15)<br>"*…what they send you when you request the* [freedom of] *information is just a joke and that is exposing how transparency mechanisms exist, but they are really badly implemented so if we get more evidence on this, we can… have people saying, 'yes, you do have it', but you just, it's not efficient… it's not what you should have… to expose that the transparency tools, existing tools are insufficient. Because they can claim they have it… it's amazing you have a transparency mechanism, but this is not as expected, when you actually use it because nobody uses it…."* (CS3)

	the role of civil society in monitoring the government and private sector (A15, A5).<br><br>To adequately monitor, there should be improvements in transparency for decision-making concerning unhealthy commodities. (A21, CS5, A14). Participants proposed improving freedom-of-information request processes (CS3, FPO2, A24) and applying them to public bodies that receive public funding or may have a regulatory role, such as advertising standards authorities (CS16); and there should mandatory and standardised disclosures for UCIs on sales (FPO4, CS16), marketing (CS16) and operations data (A1, CS14).	*"…we found, for instance, that the Advertising Standards Authority, there're a regulator for advertising, they make decisions about advertising rules, but you can't FOI* [send a freedom of information to] *them, they're a private entity. So that's just a small thing that could bring about a level of transparency. And then likewise, think tanks…. we know that a lot of companies who don't want to muddy themselves with some kind of lobbying tactics will fund think tanks like the Adam Smith Institute and the Institute of Economic Affairs to run big kind of smear campaigns against public health policy and they don't disclose their fundings. You can never prove that paper trail, even though it's quite obvious to us and it adds this kind of veneer of political support to their argument."* (CS16)<br><br>"*And that's one of the things that… Bath group [Stop Tobacco Organization and Products] is so good at. And that we need more and more and more of. It is exposing the approaches and activities in this case of the tobacco industry, in the way it works and the way it's changing… But you know, the advent of all these new funded groups and organizations. Expose, expose, expose. The more they're exposed, shine a light on them, the more difficult it is for them to pursue those approaches."* (A26)<br><br>*"Organisations can't do Freedom of Information requests because departments might not actually be recording these meetings. I found that in our own experiences departments haven't been recording the meetings, so when you do maybe find a reference to a meeting that took place or a communication, there's nothing there. No department has it, so you're missing out on vast swathes of information around the policy may have come about, and I think that's like that's really important."* (FPO2)<br><br>*"… there should be a mandatory requirement on industry to share data. And that can be done in a way that doesn't interfere with… their commercial confidentiality."* (CS14)<br><br>"[Government reports] *shows that the sugar industry was depleted due to the sugar beverage tax and the jobs were lost. They won't show us the original research, and it's not peer reviewed or that kind of thing. So, transparency is critical."* (A14)
**2.5. Provide formal and informal education or training**	Participants suggested that formal and informal education or training should be developed for issues of unhealthy commodities and UCI influence on policy, for example, in school and university curricula (CS13, A25, CS4, A16, A11, CS12), especially in business schools (A1, CS12, A7). Schools should include media literacy so that people are critical about UCI advertising practices (A13).	*"…exposing young people and students to this kind of critical analysis… so going into curriculum and education… the pedagogy, and so… for example, you know you have the schools of public health or their courses on food and nutrition. But… how many of them are really look critically at corporations, those industries industry and their influence."* (CS13)<br><br>*"Ensure ordinary people are informed/increase media literacy starting in school."* (A13-SN)<br><br>*"…have business ethics training in business schools, maybe better vetting of MBA students that are going into these schools of like high power. Not simply teaching students that "a product is a product is a product" because that's from what I've heard, a very common thing that like once you know how to sell it, it doesn't matter what the product is. But in fact, it does matter what the product is because obviously some products are highly unethical, and some are more neutral."* (A7)

	Training should be targeted at policymakers at national and international levels (GGO1, CS20, GGO2, CS12), as well as members of civil society, university staff (CS20) and judicial officers (A11) not familiar with UCI influencing practices. It should include the need for public health policies (CS20), such as for UCI marketing practices (CS14). Academics and civil society groups who are knowledgeable of UCI influencing practices should work with the media to develop public education campaigns (CS4) and develop public education on the issues around processes that enables UCI influence, such as Investor-State Dispute Settlement (ISDS) processes (A12)	*"…trainings (sic) for… early career researchers and public health people… So basically, engaging with… young people and students, and including this kind of critical analysis."* (CS13)<br><br>*"There should be an intervention around training on corporate influence. It might be obvious, but not everyone is familiarised* (sic) *with the tactics of the industry or seems as something does not affect. How? working with experts (civil society organisations, WHO, WHO regional offices, universities)* (sic)*."* (CS20-SN)<br><br>*"[P]ublic education on dispute settlement process - has proven very effective in changing the stakeholders at the table"* (A12-SN)
**2.6. Regulate the media industry and marketing**	Media companies should be mandated to disclose CoI of lobbyists and industry-funded research in media coverage (CS10) and media companies should be prohibited from providing platforms to organisations who fail to disclose funding sources (CS16). There should be restrictions on marketing of UCI products online and offline (A5, A20, CS18 FPO2), particularly to children (A13), such as France's Loi Évin style marketing regulation (A23), which prohibits alcohol and tobacco advertising on TV or cinema, and requires a health warming accompanying any permitted alcohol advertising. Regulations should also restrict the amount UCIs can spend on advertising, (A9), and there should be	"*Requirement for lobbyists to disclose interests in relation to media coverage - and for media to note them, e.g., IEA* [Institute for Economic Affairs], *Portman Group, industry-funded research.*" (CS10-SN)<br><br>*"… anyone that's taking money from these companies should be required to disclose it… and the…* [media companies] *should stop giving them* [industry funded think tanks] *a platform if they're not disclosing their funding…"* (CS16)<br><br>*"…if you allow any kind of grey areas then it is hard to prove, it is hard to enforce, it is hard to monitor it. It makes a lot of complications, especially with our modern digital marketing technologies, social networks…" (*A5)<br><br>*"…an Loi Évin approach to marketing regulation… that's a really good example actually of a concrete policy initiative that… multiple actors across different industries different sectors could get on board with. So, it's a concrete empirical change in policy or change in legislation that has a precedent… we don't like kind of parallels to Europe in the UK anymore. But it's in a closely neighbouring country and there's a rationale for it. And there's evidence of its effectiveness as well."* (A23)<br><br>*"Massive spending on advertising/false charities… "sells" positive image of industry - no caps on expenditure."* (A9-SN)

	adequate enforcement mechanisms for such regulations (CS18).	*"Strict marketing regulations of products in laws and online - working with social media platforms to enforce their own regulations."* (CS20-SN)

Table S3. Interventions to reduce UCI ability to prioritise their growth and profits

Intervention category	Intervention description	Illustrative quotes
**3.1. Challenge neoliberalism and GDP growth**	Participants suggested challenging the fundamental assumptions of neoliberalism (A10, GG01, A4), and adopting degrowth or wellbeing economies (A3, A4, A17, A20, A2) by applying alternative measures of economic growth (A4, A3) that embed public health (CS11, A22, A7), equitable distribution goals (A3), and account for the depletion of the natural environment (A10). For example, Bhutan's Gross National Happiness Index, which seeks to achieve a level of happiness and wellbeing in their development goals (A17). Participants also suggested incentivising corporations to aim for positive impact on the environment and social, health and employee wellness (A1, CS11), and develop greater benefit sharing mechanisms (A3) and debt forgiveness (A3) in international development practices.	*"…the nuclear option is to remove competition… if you remove competition, then you remove this need for companies to really prioritised profits over everything else and forward the whole system to prioritise profits and accumulation of wealth over everything else. Then you're talking about it like post capitalist systems* (sic)*."* (A4)<br>*"…deployment of alternative discourses. So, I mean just feeding on the points we've said before about how the kind of rhetoric of neoliberalism..."* (A10)<br>*"…one argument that economic growth as currently constructed, if we're going to come at this from a green and environmental model, we actually need to see the end of growth."* (A22)<br>*"Challenge the GDP growth/economic growth discourse (see Bhutan)"* (A17-SN)<br>*"…Norway ostensibly has its well-being economy principles in place, because of that, they* [states] *would have other particular metrics that they would want to have ticked for that and they would have slightly different priorities coming into that trade negotiation."* (A2)<br>*"…the well-being economy is that it's simply to say that GDP shouldn't be the only metric of success and as you say others* [metrics] *should be pursued in parallel and complementary to that…one of the other criticisms is that GDP is at the national aggregate level, and it says absolutely nothing about inequalities and inequities at the population level. So, I feel a more equitable distribution of the GDP would perhaps be part of that…"* (A3)<br>"*…redressing the wealth extraction from South to North through benefit sharing mechanisms, sovereignty, etc. …"* (A3-SN)
**3.2. Reform investment practice**	International standards on Socially Responsible Investing (SRI) or Environmental, Social, and Governance (ESG) investing should be developed for individuals or insurance companies (CS17, CS1, A1, CS2, A26, A7). Participants suggested that investors should undertake Social Impact Investing and invest in Impact Weighted Accounts (A1, A7) and	*"…within lending and investment activities… there's different investment strategies or whatever now called the ESG investments, Social Impact Investing. There's a new thing called Impact Weighted Accounts and they're all sort of different in their own way, but they're new mechanisms that theoretically are trying to be used to change investment practices, but they're also in some ways still pro-industry mechanisms."* (A7)

Intervention category	Intervention description	Illustrative quotes
	governments should invest in small and medium enterprises instead of large corporations (A22).	*"…the United Nations is really trying to put all these* [ESG scores] *together under this UN principle of responsible investing. So, this is sort of the tool I would say, or this would be the way to go, ultimately when you want to come sort of send a signal to the corporation."* (CS17)<br><br>*"…you're not contributing to economic growth by consolidating the power* [of multinational corporations] *in capital. The way you… get economic growth is if you invest in small and medium enterprises and networks of them….* [investment] *is really key here because the role of the state is to protect the powerful too often, whereas… in some countries… their role is to promote small and medium enterprises…. this is where it comes down. This is where I do think power is important here. Because the role of the state can be to protect these big corporations, or it can be to be disruptive and support growth from small and medium enterprise."* (A22)
**3.3 Reform corporate ownership and management**	Corporate charters and corporate legal entities should be changed to include social and environmental impacts as corporate goals, for example, as some participants suggest for Benefit Corporations (A7, A1, A3, A25). Rules on corporate fiduciary duties should change to include social and environmental stakeholders (A7, A1, A25, CS9), remove corporate limited liability (A3, A25), and mandate direct liability (CS18, CS19). This would allow for corporate owners or management to be liable for company wrongdoings (A23, CS2), such as for violations of human rights and environmental standards (A12, CS1). If significant corporate harm is found, there could be judicial dissolution of, or restricting, the corporate entity in certain jurisdictions (A3, A23, A24).<br><br>Alternative forms of business structures should be developed. This may include co-operatives that mandate worker participation in company decisions (A9) or establishing government-owned manufacturing and/or retailing companies for unhealthy commodities, for example, modelled on Canada's Provincial Liquor Crown	*"…Benefit corporations, it's like a new type of corporation. It's in several different countries now where corporations can pursue both a social purpose and profits and within benefit corporations, that duty is extended to all of their stakeholders rather than just the shareholders. So, I think if you were to say, for instance, to change that or modify that for all corporations to include additional stakeholders then you would get away from that problem where public corporations can just say 'well, we have a duty to our shareholders'."* (A7)<br><br>*"I think one of the major issues is the role of the state deciding the norms of how corporations can operate. So, if you don't put some limitations at that level, we'll be working forever without... achieving some… success in some particular areas..."* (CS9)<br><br>*"…creating sort of personal individual obligations for the kind of directors, management, and ownership of companies… you take the idea of legal personality seriously and you apply it both ways. So, obligations as well as rights. The other approaches that actually corporations are collections of individuals or whatever… So, if you're the chief executive of your organisation and something happens on your watch, then you're on the hook for it. And you know the idea being that it would make people have far greater oversight or be less willing to take risks around that kind of stuff."* (A23)<br><br>*"…the concept of corporate death penalty, which is that corporations have personhood in a lot of ways, so they should be able to be legally dissolved. They should have a death penalty for extreme cases, but it's never really been acted upon…"* (A24)<br><br>*"Develop alternative forms of organisation for business e.g., co-operatives - that are mandated to involve workers and are accountable for their health impact."* (A9-SN)

Intervention category	Intervention description	Illustrative quotes
	Companies or the Vinmonopolet (i.e., Norwegian government-owned alcoholic beverage retailer) to encourage fair competition (CS14). Moreover, there could be remunicipalisation of some public services (A3), and mandating diversification of corporate boards (A1).	*"...norms on legal corporate entity, corporate limited liability, norms on legal corporate legal entity, and the role of civil society in all this process of corporate accountability*" (CS18-SN)<br><br>*"...dealing with the state monopoly is like a dream. If your product meets the production standards, they have to stock it… in the Norwegian state monopoly…. to sell beer in Tesco, you have to pay. You have to rent shelf place from Tesco… to get your beer on the shelf, so… the state monopoly is, it's much better* [i.e., fairer] *for a small producer to work with*" (CS14)
**3.4. Create fair competition and encourage the production and consumption of healthy alternatives**	Participants suggested that UCIs (A7, A25, CS6, A21, A22) and media industry monopolies (A2, FPO3) should be broken up. Governments could support newcomers of healthier products or services into the market to compete with UCI incumbents (A4). For example, they could support smaller local businesses (A22), changing rules on subsidies to UCIs, (A1, A8), establish alternative forms of business structures (CS14), or providing healthier alternatives to consumers (A1, A10), through food aid programs (A8) or universal basic income (A3).	*"If consumers stop choosing products from unethical companies, then these unethical companies will be forced to change or will fail. A big problem with this is monopolies and the ubiquity of unethical behaviour of companies can make it difficult to choose more ethical options."* (A7-SN)<br><br>*"...in climate change we're facing the issue that we have an incumbent industry that we need to wind down then we need to… completely eliminate fossil fuel for the future emissions, and the industries that are implicated in the extraction, production and consumption of fossil fuel emissions. But at the same time, we need to scale up an alternative… and new industry that would replace the energy services, so those companies currently deliver. So, it's the…transitioning from…one regime to another."* (A4)<br><br>*"They're* [governments] *governing in the interests of the large corporations, not their people… if they were then they would be creating a thriving economy in which anybody could start up a small company. They could function locally, they would employ people locally... What they've done is they've allowed the consolidation of power in the hands of a small number of people who are exploiting workers in the producer industries, farming and things like that."* (A22)<br><br>*"...making the impact of corporations on health known and providing other choices."* (A1)<br><br>*"...the national food strategy in the UK… which came out last year also used various discussions with…people up and down the country in the UK showing… people saying you know we just can't afford healthy food...*" (A10)<br><br>*"To combat privatisation: Build public sector as effective alternative to private sector, e.g., institutional food programs, public food benefits, subsidies to small farmers instead of agribusinesses."* (A8-SN)
**3.5. Minimise externalities, reform taxes and**	Governments should minimise externalities through mandating true cost accounting (CS9, CS1, A1, A9), and introducing 'polluter pays principle' (A20) by taxing UCIs (CS11, CS6, CS10, A13). Importantly, there should be:	*"...when I think about the overall structural issues, the only way* [of] *focusing on NCDs and its risk factors, it's like if we don't start having like true cost accounting, we'll never end this, because I mean you're allowed to profit and some products that are killing people, killing the environment. It's bad for our health. It's bad for our planet…"* (CS9)

Intervention category	Intervention description	Illustrative quotes
**invest in the public sector**	improved enforcement, collection, monitoring, and oversight of existing tax laws (CS6, A1, A25); the elimination of corporate tax avoidance (A25, A9); the closing of loopholes for tax havens, evasion (A4) and tax deductibles (FPO4, A3), and tighten controls on corporate tax labilities (FPO4). International regulations for tax (A4) could be developed, such as a global consensus for corporate tax baseline through the United National (UN) (A3). Departments for health could be mandated to have control over taxes that impact public health (FPO4), such as setting (A20) or earmarking taxes on unhealthy commodities to use for public health services (A22, FPO4, A15) or establishing an independent health promotion foundation, such as with Thai Health Promotion Foundation (A14, A15).<br><br>Governments should institute progressive and redistributive taxation (A3, CS13, FPO3, CS6) to adequately fund public services international governance institutions (CS10, A12, A15, A6, A22, FPO3, CS13) so that they do not need to rely on PPPs or private sector donations (CS13, A8, A12, CS10, A6, FPO3, CS9). This would strengthen public strengthened to better protect and promote public health interests more (CS6, A14, CS13, A15, A6).<br><br>Governments should provide funding to implement regulations for UCIs (A13, A22, CS3, CS11, FPO3, A6), including the funding of public regulatory bodies to enforce regulations and (A1, A13, A14, CS13, CS3), provide counter advertising for unhealthy commodities (A13, A14) or education campaigns explaining how	*"…there are transnational corporations that are actually causing harm. Their footprint is negative for our societies. And I think that has to be drawn back, so I think that is a globalisation conversation to put globalisation more back to the people, to the community and for us taxation is very interesting…* [a participant] *called it externalisation. We are socialising the harm that these companies cause. My parents pay for it, but then they go home, the shareholders, the business executives with windfall profits. So that has to change."* (CS6)<br><br>*"Introduce polluter pays principle for all unhealthy commodities industries and spend funds for health promotion and other public health measures."* (A20-SN)<br><br>*"Strengthen institutions (tax collection, oversight) + global tax justice."* (CS6-SN)<br><br>*"Strong enforcement (Tax)."* (A1-SN)<br><br>*"Tighten capacity for TNCs to avoid tax."* (A9-SN)<br><br>*"Something else… is probably tax havens, tax avoidance, tax evasion… the most environmentally destructive companies are those that are the most offshored companies. There are also the companies that have the largest global influence because they're multinationals, they're incorporated in multiple locations, and they have their money protected in different arrangements. That's more complicated because that's an international issue so that requires not domestic regulation but international regulation."* (A4)<br><br>*"[P]rogressive taxation, solidarity taxes, wealth taxes, UBI* [universal basic income] *etc (sic)"* (A3-SN)<br><br>*"I believe that… taxation, either general or selective… essentially what that is all about is using the power of the state to make sure that more of the resources… are returned to the countries in which they're doing business. That's one thing so that you're actually stopping that capital flight in some way so that there's more money for public goods. And the second issue is so that's more about raising money for the state in different ways."* (A22)<br><br>*"…it's about budgets and financing and strong public systems and a strong you know government… So, you have a bureaucracy which is well funded…. and by bureaucracy, I don't mean only the people at the ministry level… but… the whole public system. And I think a lot of the problems…comes… due to… lack of investment into public institutions and… public services and systems…so… having more budgets for public institutions for government, for public services."* (CS13)

Intervention category	Intervention description	Illustrative quotes
	health policies will be effective (CS4). Such investment could also develop the skills of public sector officials so that they understand how UCIs influence policy (A9).	*"…the important things financial and funding wise for public spending that go on in governments…. probably you need some kind of [tax] mechanism for anything with public health implications to give the Department of Health some kind of lever, which they don't have at the moment."* (FPO4)<br><br>*"…advocating for many of these things takes time, money, and research to back up evidence. And for me… using tax money to create a health promotion foundation in order to enable this to happen more easily without conflict of interest is such an important step that could be taken and has been taken in many countries."* (A14)<br><br>*"…proposal for UN consensus on global corp tax baseline."* (A3-SN)<br><br>*"…you know progressive taxation… that's another thing that the industry constantly sort of tries to scuttle so…your CSR, your philanthropy, and also… you become like a benefactor… you have this kind of very, 'charity' kind of relationship. But even there we see, you know, growing philanthrocapitalism that is evident not only at the global level but at the country level."* (CS13)<br><br>*"…there's been a proposal for government just to send more in, like basic contributions that are not marked for specific issues, and the proportion that WHO of funding* (sic) *that they get that isn't earmarked and that comes from Member States at all has just dropped off a cliff dramatically over the last 20 years, meaning that WHO have to go around with the hat to kind of get earmarked contributions from governments or get more and more from external entities* [private philanthropic organisations]*…"* (CS10)<br><br>*"Resources for implementation is the biggest challenge, and whether that is in terms of… human resources versus skill sets versus finances… that would always be a challenge."* (FPO3)<br><br>*"…the strength of institutions and the capacity of institutions to respond to the public interest. So, I think that is very important in this whole conversation. There has been the playbook of the neoliberal ideology to undermine the functioning of institutions in the developing world in the 80s and 90s. But we can see that when the alcohol industry doesn't like a regulator… they attack the regulator, they undermine the functioning of that institution."* (CS6)<br><br>*"Use tax money to tackle/research/counter advertise to deal with industry marketing and issues - e.g., in a Health Promotion foundation* (sic)*."* (A13-SN)<br><br>*"[I]nvest in strong, skilled public sector who understand the dynamics of industry influence."* (A9-SN)

Table S4. Interventions to reduce UCI ability to leverage legal and dispute settlement processes

Intervention category	Intervention description	Illustrative quotes
**4.1. Reform Investor-State Dispute Settlements processes and judicial proceedings**	To help curb UCI ability to leverage legal and dispute settlement processes, participants suggested reforming ISDS processes by amending World Trade Organization (WTO) rules (A3, A10). ISDS processes should be changed to allow for the establishment of mechanisms for some forms of arbitration to be dealt with in domestic courts (A12), reduce arbitration costs for governments in domestic courts (A8), mandate greater arbitrator diversification in ISDS courts (A1), and cap compensation in court decisions, for example, based on a country's GDP or income level (A12).<br><br>Participants also suggested developing stronger public health protections for UCIs in WTO rules (A10, A1, CS6, A18), such as public health clauses (A1), or exempting public health protections from litigation (A10); the 2001 WTO Doha Declaration, which sought to balance protecting intellectual property rights and ensuring access to essential medicines (FPO1).<br><br>Domestic resources should be mobilised through the taxation of externalities to fund government legal defence in ISDS processes (CS6). There should be mandatory disclosure of CoI for judicial officials and arbitrators (CS20) and recusals in ISDS proceedings where CoI are found (A12, A25, CS19).	*"[N]o ISDS in trade agreements (some countries are already going down this route)."* (A3-SN)<br><br>*"Stronger public health protections in trade agreements (eg exemptions from litigation); ISDS.* (sic)*"* (A10-SN)<br><br>*"Introduction of singular forum for disputes involving corporate conduct (currently, disputes from corporation can go to ISDS; disputes against corporation needs to go to domestic system)"* (A12-SN)<br><br>*"[G]reater diversification on… dispute settlement courts…"* (A1-SN)<br><br>*"…when that changes* (sic) [government beginning to tax negative externalities], *the domestic resource mobilisation changes it. I think then governments can all of a sudden afford* [to undertake] *some settlement* [ISDS] *process… you avoid the regulatory chill effects. The industry then loses, they can pay for the lawyers, low-income countries cannot so, but many things improve if the tax base improves…"* (CS6)<br><br>"*The Doha Declaration… that was low-income governments pressuring the… World Trade Organization… was a variation on a TRIPS agreement… the Doha Declaration refines what is in the trade rules as we could call them by saying that in certain settings in certain countries for public health purposes, this would happen to be AIDS, intellectual property rights do not necessarily dominate.*" (FPO1)<br><br>"*…stronger public health protections as kind of binding clauses within trade agreement... for example, through exemptions from litigation and also, you know, not just through explicit public health protections, but the dilution of the corporate protections that are then used to contest the health policies. So… the ISDS clauses being, you know,one of the main contentious issues in this space."* (A10)<br><br>*"…if authorities or these policymakers understand this conception* [how UCIs influence policy] *this will also allow them to understand that the industry or corporations interfere in different layers and that* [UCIs] *do not only target the ministries of health... For instance,* [an intervention could be for] *court law authorities* [judicial officials/arbitrators] *to implement mandatory codes of conduct to disclose conflicts of interest in international trade…"* (CS20)<br><br>*"Capping reparations* [compensation awarded in ISDS tribunals] *or tying it to a state's GDP and other obligations can lessen impact of leveraging dispute settlement system."* (A12-SN)

		"…mandatory standards for recusal of judges in situations where that doesn't exist (US Supreme Court; investor-state dispute) can be disruptive to same issue (sic)*."* (A12-SN)
**4.2. Undertake Strategic litigation**	Participants suggested bringing strategic litigation against UCIs or governments (A10, CS5, A17, CS7, CS4, CS17), by leveraging existing consumer law (A1), especially on misleading information (CS1). Making changes to the status of corporate legal entity, such as limited liability, would support strategic litigation (CS17).<br><br>'Victims' could bring cases against UCIs for their attempts to influence policy and encouraging the consumption of unhealthy commodities (CS5). Litigation or disciplinary hearings could also be used to enforce policies such as, CoI policies in decision-making (A15). There should also be international cooperation between governments and civil society to support each other when UCIs litigates against government (CS20).	"*…what seems to have contributed so enormously to the delegitimisation of tobacco involvement was what came out as a result of strategic litigation against the tobacco industry. So that kind of specific action, strategic litigation and everything that follows from it, I think, I mean obviously that's not easy to do. But that seems to me to be… one thing that at least* [that] *reconciles and encompasses and leads to both the top down, bottom-up changes…"* (A10)<br><br>*"…aim to mobilise the grassroots, a civil* [law]*suit from civil society in general or the doctors or patients, or youth* (sic)*."* (CS5)<br><br>"*…aside from trade litigation or sort of investment related litigation, the main litigation… like the Master Settlement Agreement of the US that… arose from litigation based on corporate wrong, based on tort. The ability of the US to file that kind of a case, and the ability of many high-income countries to file that kind of a case has a role to play in corporate behaviour… that should be included because that's corporate liability…"* (CS17)<br><br>*"[H]igh profile examples of regulatory/enforcement actions (e.g. court cases/disciplinary hearings) to rectify COI"* (A15-SN)
**4.3. Fund legal practices to oppose UCIs**	A WHO fund could be created to provide legal support for governments undertaking legal action against UCIs (A19). A public interest legal fund could assist whistle-blowers and governments in defending against litigation by UCIs or to engage in arbitration processes (CS18, CS20, CS6). Participants suggested mobilising domestic funding, such as taxes, to fund civil society legal actions (CS20), and proposed that philanthropies who fund industry monitoring organisations could also fund organisations' legal activities (A26).	*"WHO fund for legal support for governments/advocates."* (A19-SN)<br><br>*"…we've talked about for a long time is like a sort of… legal pop fund for governments and whistle-blowers. So, if you're going to play the whistle on the industry, or if you're a country that's… like a poor African country that's going to implement a health law and you know you're going to get sued by the industry… there's a fund that helps you out. So, you can actually stop the industry delaying legislation."* (CS18)<br><br>*"Domestic resource mobilisation determines ability to engage in dispute settlement."* (CS6-SN)<br><br>*"Encourage major charities to fund work on advocacy and exposure of industry tactics - needs resources also need funding for legal activities."* (A26-SN)<br><br>*"[F]inancial support to create capacity. How? Through litigation itself, through taxes, trough international cooperation. Impact: Corporations think twice before suing a State or a specific policy* (CS20-SN)
**4.4. Leverage human rights**	Legislation should be developed mandating human rights and environmental due diligence	*"…we're seeing a sort of push for it* [mandatory due diligence] *to become an EU Directive on that issue, and once the EU adopts it then the UK will have to do something… But we've already started to*

	for supply chains, and the direct liability for violations of human rights standards by corporations at the national and international levels (A12, CS1). Civil society and governments should utilise UN reporting mechanisms for human rights abuses by business (A13).<br><br>Government's human rights obligations should be leveraged, such as the right to health (A14), benefit from science (A13), access healthy environments (CS15) and public participation (A13, A4). This would help to prioritise social and public health goals in national and global level decision-making (A10, CS3, CS4, A1) and encourage greater civil society participation in policy processes (CS3). Human rights goals should also be enshrined in international trade and investment agreements (ITIA) (CS6).	*see a coalescence around that and what it does is fundamentally shift the burden of responsibility so it disrupts the issues that arise from corporate legal personality and from the limited liability of corporations, and it disrupts that and pushes what corporations have traditionally been able do, which is push down their supply chain and their responsibility so the companies with the least amount of investment in the least amount of income are the ones who are actually bearing the greatest burden and it inverses that and pushes it back up to those who have the greatest income.*" (A12)<br><br>"*…the right to enjoy the benefits of scientific progress… it's not well developed, articulated, and there's opportunities to say, 'well, actually every state has to report every five years to this UN committee. You should be reporting on how you've tackled these problems because science is a victim of corporate influence in this context'… there's no public participation in there, and the scientific implications is potentially serious so."* (A13)<br><br>*"…you need to have a discussion on how you design those public policies and… the best way to do that is deliberative, like public participation in the design of those policies."* (A4)<br><br>"*…there is an obligation on states to ensure that people benefit and enjoy the benefits of science scientific progress as it's framed so you know the question of funding, the health promotion programs or research into health promotion should also be a state obligation*." (A13)<br><br>*"…there are also other powerful ideas like human rights-based discourses, social justice-based discourses… certainly they can potentially be powerful to deploy as* [a] *kind of counter to their traditional economic arguments."* (A10)<br><br>*"…the arguments about human rights, the human rights to water, to food, to… access to all these things, has been a very big argument done* (sic) *to contest any neoliberal argument that, you know, industry have rights as people... It's getting stronger and I think it's a way… to get to success to be honest. So, this has been the main way to… in the WTO level… contest any decision. This has been the main argument against, for example, front of package labelling and warning labels in food. So, this is a big thing that should, I think, it's a good tool to reduce interference of corporations when decision-making"* (CS3)<br><br>*"[There is a] [s]eparation between trade - health - human rights policy arenas >> limited chance to hold alcohol industry accountable within trade arena since health and human rights are absent."* (CS6-SN)
**4.5. Develop and leverage international treaties**	International treaties comparable to WHO FCTC for each unhealthy commodity should be developed which would include an Article 5.3 equivalent (CS6, FPO1, A2, A1, CS7, A20, CS4, A15, CS17, A26). Participants also suggested developing other treaties, such as a Framework	*"…there is work going on at the Human Rights Council for a global binding treaty to hold corporations accountable for their human rights abuses. And we are working for a global binding treaty on alcohol…so… you mentioned the Article 5.3 and the FCTC. And we need something like that for alcohol."* (CS6)

	Convention on Global Health, based on the right to health (A14, CS7), international regulations for corporate tax baseline (A4), or an international treaty on business and human rights (CS6, A9, A8, FPO1, A13, A11) with strong built-in enforcement mechanisms (A9). Such treaties could be used as counterweights to ITIAs, especially in ISDSs. Implementation and adherence to any existing and future treaties could be improved through close coordination between international governance institutions and national policymakers (CS20), especially the WHO FCTC Article 5.3 (CS17, CS7, A20, CS20, FPO1, CS18, CS20).	*"We're much better off with one than without one [WHO FCTC]. We've learned a lot about [Article] 5.3. if we* [are] *starting afresh* (sic), *we'd do it differently…"* (A26)<br>*"…different industries use the same playbook. So, we need to be supporting each other more… in looking at the commercial determinants* [of health] *and how to leverage information like we're talking about a convention on alcohol or should it be a Framework Convention* [on Global] *Health..."* (A14)<br>"*…what Article 5.3 has done is strengthen the spine of several governments to actually hold the corporations to account… Push for treaty negotiations where you can get them. And then if you have a treaty under negotiation, absolutely push for something that is the equivalent of Article 5.3 of the FCTC. It's foundational."* (FPO1)<br>*"…I have this sort of an Article 5.3 mindset, so I probably would suggest, like all the interventions I have in mind are… all the eight recommendations that you see in Article 5.3 and those would be the interventions. And especially that's crucial… l would plug each and every of them in one of these* [themes]. *"* (CS17)<br>"*…national and local level is… where things should happen. And so, if there was…*[an] *international instruments* [that] *really empowered national and local governments somehow to be able to enforce the provisions of treaties or local laws, I think that would be really important.*" (CS7)<br>"*…an international instrument that deals with business and human rights… the idea is to sort of expand international law and jurisdiction to also be able to hold certain international corporations to account."* (A11)<br>*"That's* [tax havens, tax avoidance and tax evasion] *more complicated because that's an international issue so that requires not domestic regulation but international regulation"* (A4)
**4.6. Develop anti-corruption legislation**	Participants suggested developing anti-corruption legislation to make political practices UCI use to influence policymakers a serious criminal offense (A11, CS5). Such legislation would cover UCI lobbying, political financing, revolving door practices, transgressions regarding CoI, and making bribery by UCIs, as well as provide protections for whistle-blowers (A17, A15).	*"While the issue of actual legal corruption where, I mean it is very different from context to context, but it does speak about sort of industries' ability to circumvent their legal obligations, that is also important and sort of the intervention for that would probably be very different and would look at a lot of different things."* (A11)<br>"*Those cases of where the industry uses corruption as a tool to basically what they do they give bribes, or they make regular payments. Let's say to certain civil servants for them delaying the processes not doing their job properly…. they do manage to build that relationship and to have that public image and public relations that they are never caught…."* (CS15)<br>*"Effective whistle-blower protection which we do not have in…* [our country], *except in name only"* (A15-SN)

Table S5. Interventions to reduce UCI ability to leverage policymaking norms, rules, and norms, rules, and processes in their favour

Intervention category	Intervention description	Illustrative quotes
**5.1. Reform government regulatory frameworks**	Health impact assessments (HIAs) should be mandated for national and international policymaking (CS3 CS7, A1, CS4, A10, A2), including for ITIA (A1, A2) and industry self-regulation (CS14, CS12, FPO4). HIAs should include the economic costs of public health problems (CS11) and the benefits of regulation for population social life, health, and well-being (CS10, CS15). Finland's Health In all Policy approach was noted as a good example for considering the public health implications of policy decisions (CS14). In addition, participant suggested that accepted standards for the assessments of public health risks of products should be developed and introduced for regulating UCIs (CS18).	*"...this kind of assessment you have to do of new regulations to demonstrate its going to be low risk and it's going to be cost effective. Those rules don't apply to self-regulation…. Self-regulation usually involves self-assessment of your impact as well…. It kind of relates to the ideology that if it's self-regulation it's not a burden so therefore there's no need to assess it. But the issue of effectiveness tends to be overlooked."* (FPO4)<br><br>*"… I think it's necessary to, on a more general level, to elevate the public health interests and standing or public health… through mandatory… health assessments. 'Health in All Policies' way of thinking, which is a whole line of thought in Finland, has been very much promoting that way of thinking…"* (CS4)<br><br>*"Mandatory health impact assessments of all TIAs* [Trade and Investment Agreements]*"* (A1-SN)<br><br>*"Requirement for genuine health impact assessments --> HIAs* [Health Impact Assessments] *that are reflected in policy & lend to accountability measures"* (CS7-SN)<br><br>*"...you could consolidate recommendations about reforming trade and investment agreement negotiations e.g., transparency, health impact assessments…"* (A3-SN)
**5.2. Reform policy consultation and deliberation processes**	There should be formal rules, such as parliamentary procedures, on how public officials should engage with UCIs in policy consultations and deliberations (A6, CS11, A17, A2, A15, CS9). Participants suggested requirements for public and civil society representation or participation in policymaking, (A9, CS5, FPO3, CS13), for example, deliberative policymaking processes at national and international levels (A4, CS8, A22, CS7), including in ITIA negotiations (A10, A12), the WTO (A9, A2, A3), and global governance organisation, namely the World Bank and UN agencies (A3, A9). A participant noted these approaches have successfully applied to climate policy (A4). Standards should be developed for	*"You need the supportive policy level changes – case studies, these things need to be formalised and institutionalised and have parliamentary rules so that they* [UCIs] *are not part of* [policy] *deliberations."* (A6-SN)<br><br>*"Single coherent set of rules for all parts of government to engage with industry in policymaking process. Ideally across industry but could focus on big industrial harmers* (sic). *Likely challenging to implement nationally in the current climate but could piloted at local government level…. Lobbying, consultation, partnership etcetera. PHE developed a set of rules before being disbanded - no reason why they couldn't be expanded to all parts and tiers of government."* (CS11-SN)<br><br>*"… you need to have a discussion on how you design those public policies and… the best way to do that is deliberative public participation…and from a broad cross section of society and you say, 'right, your job is to take in the evidence, speak to the scientists, speak to the policymakers and design us a kind of policy that will deal with this problem'… So, across the political spectrum across level of expertise and parts of society that they may be representing, they achieve consensus and they put forward proposals that makes sense… So, if you design a policymaking system that basically it has that format – did they have this deliberative form that includes experts that includes stakeholders? And then they provide a recommendation on that."* (A4)

	the inclusion of evidence in policy consultation and deliberation processes (CS3, CS9, A15).	*"…it's about introducing participatory governance at all levels, whether it's local, government, regional…state, international organisations… But you know, even the local government, there's lots of ways that industry can influence planning decisions, of course, and right through the system. And we've always struggled… with participatory governance. But it's really important. You actually have structures where people without a huge vested interest (sic) actually making the decisions. Civil society of course, a free and open civil society plays a big role in that…"* (A9)<br><br>*"Improve standards of 'evidence' and transparency of it to be considered for policy formulation."* (CS3-SN)<br><br>*"I see how it happens* [industry submitting comments for policy] *and I also put one comment that it also diverts a lot of civil servants, technical areas time* (sic) *because they have to respond all the time to the industry requirements"* (CS9).<br><br>*"… with tobacco victims it was a challenge and I guess it still remains a challenge of having those people* [victims] *being part of the campaigns, raising their voices, you know and that's what made our work harder because we are the advocates, we are there speaking* [in policy consultations and deliberations] *on their behalf"* (CS5)<br><br>*"… at* [the] *World Trade Organization you could make an argument for more civil society attendance… industries are always invited, and they have multiple… industry roundtables and discussions and sometimes civil society are just outside or public health experts… So, there's like a big disparity in that…"* (A2)<br><br>*"Open Government Partnership, which is at the sort of national and state level but do that and ensure that it gets then applied to trade ministers and to trade… delegations and try to use that as a model to embed more civil society participation for those delegations."* (A3)
**5.3. Represent public health interests in governance institutions and policies**	Public health actors should be represented in trade regulation activities (A7), including ITIA negotiations (A1, A10). More power could be given to health ministries (A19), such as ensuring that departments for health lead on policies with health implications (FPO4, CS14). Governments could also develop strategies to achieve policy coherence (CS6, FPO4), such as instituting inter-ministerial committees for public health, issues, reflected in Finland's Health in All Policy Approach (CS4).	*"…instituting inter-ministerial committees which have public health as their main prerogative or main task could be one way of doing it to make public health a more important part of the government decision-making processes…"* (CS4)<br><br>*"…what we were talking about with trade, you could include public health professionals as equal partners to industry during trade processes and increase awareness amongst the government that this is important."* (A7)<br><br>*"…enabling public health input on equal footing with industry. So that's in the design of trade agreements… And it's very difficult to get ones' access in the UK, for example, to come to any of the kind of negotiating documents in the various trade rooms, we're negotiating, but industry does have access to those documents and also opportunity for comment through various trade committees. As well as being seen as an important stakeholder by government."* (A10)<br><br>*"… the Home Office led on the policy* [minimum unit pricing in Scotland], *so they led on it for crime*

		reduction reasons, and not for health reasons, from their own point of view. And I do think that was an ingredient in why it didn't come in. So, it just wasn't as important to the Home Office as it would have been to the Department of Health." (FPO4)<br><br>*"Make sure that particular government department lead on particular polices that have health implications."* (CS14-SN)
**5.4. Develop conflict of interest policies at all levels of governance**	Rules for personal, institutional or financial CoIs should be developed for national governments and international governance organisations (CS10, CS17), including decisionmaker dealings with the private sector (CS10, CS8, A15), declaration of owning UCI stocks and shares by policymakers (A16, A10), and the removal or recusal of public officials from working on policy issues if CoIs are found (A25, CS19, A12). Participants noted that generally CoI policies were not well understand or conceptualised in governing and research institutions (CS3, CS10, CS12, A25). There should be mandatory disclosure of CoI for policy actors in policy consultations or deliberations, such as industry representatives or third parties, including consulting firms or civil society groups (A3, CS11, GG02, A26).<br><br>For individuals or organisations representing UCIs (e.g., corporate officials, producers, marketers) that have a personal, institutional or financial CoI in a policy area, there should be mandatory restrictions on their participation in formal or informal governmental committees or policy discussions and consultations (A25, A23, A14, CS16, FPO4, A2, CS12, A1, CS20, A15, CS19, A12, CS6), and there should be sanctions and enforcement for violations of CoI rules (A14, A25). Governments should create a governing body to decide whether a CoI exists in a particular instance (A16, CS12), monitor public officials for CoIs (A5, A25) and provide capacity	*"WHO is increasingly being asked by governments to provide guidance on just identifying conflict of interest… obviously that does exist to some extent in tobacco, but it doesn't exist for junk food, doesn't exist for alcohol, and actually then they're out of sync with each other. So, where WHO may have slightly better processes and red flags for who they can engage with that might be undermined by those entities being able to engage with the United Nations Development Program, for example, or whether there are more restrictions at the kind of the global level they might be able to engage with the UNDP national officers in some countries…. There's like every loophole, that can be exploited, there really is. Also, to the extent that, you know, WHO recently took a donation from Nestle, even though it's completely against their own internal rules and the international code on breast milk substitutes."* (CS10)<br><br>*"[A]t national level - governments using COI when assessing industry attempts to get onto committees/influence policy…"* (A2-SN).<br><br>*"Conflict of Interest safeguards + Transparency + Requirements for policy coherence."* (CS6-SN)<br><br>*"…we really need a lot stronger policies between conflicts of interest, this may be mentioned before, between governments and industry and why are we allowing…* [industry]*… for instance, to influence the CDC?* [Centre for Disease Control] *Why is it happening?"* (A7)<br><br>*"…some of the CoI is pretty damn hopeless. And so, you get quite a lot of people who work for the industry who just don't declare their conflicts"* (A26)<br><br>"*WHO is increasingly being asked by governments to provide guidance on just identifying conflict of interest...*" (CS10)<br><br>*"[R]igorous COI standards that go far beyond tobacco exceptionalism to include e.g., consulting firms…* [philanthropic organisations] *…"* (A3-SN)<br><br>*"If… every cabinet minister had to declare their potential conflict of interests, what would that look like? How many family members have tenders? How many…?... you have to declare that as a potential* [conflict of interest] *and then there should be a committee or something that then decides on that or their rules to govern it..."* (A16)<br><br>*"…increase the requirements for on conflicts of interest requirements in the reporting and monitoring enforcement on members of Congress. The staff of Congress. On Judges. And at the federal and state*

	building for decisionmakers around issues of CoI (A14).	*level, governments all… through government. Our requirement strengths and the requirements for disclosure, the requirements for recusal. There should be some prohibitions on appointment of corporate officials. And extend the amount of time between when they leave government and when they can contact government."* (A25)
**5.5. Change practices on multistakeholder governance**	Participants suggested changing practices on multistakeholder governance that promote UCIs as legitimate governance actors involved in policymaking (A23, GG01, FPO2, CS14, A24, A9). Advocates should seek to counteract UCI involvement in policymaking processes (CS14), particularly at international governance institutions, such as the WHO (GG01).	*"So, they* [UCIs] *claim the rights of people and protections from government, but they also participate in governance, and they create all sorts of obligations on individuals, and they shape our experience in society as individuals and exercising almost power over us individually… is really quite outrageous actually."* (A23)<br><br>*"… you're* [UCIs] *giving a semblance of autonomy, of moral autonomy and authority that isn't real. It doesn't exist. But you're giving that semblance as a means to gain status as a legitimate partner, as a well-intentioned social actor. And all these kinds of things, when in reality you're just a business that does business for business"* (A24)<br><br>*"I mean, the big question that comes to mind for me is… how industries' participation becomes delegitimised…. part of this is about the institutions and structures that give corporation a seat at the table, but also the fact that that's seen as legitimate…. both among the public and civil society and among policymakers."* (A9)<br><br>*"…there was a kind of understanding that you know this* [alcohol policy] *was going to be a bit of a battle and I used that kind of language of conflict quite deliberately. So, I think previously the problem had always been one of partnership, partnership, partnership where we do need to be in partnership. So, I think we* [public health advocates] *decided… that we really need it to be clear we're not in partnership here… there might be some things that you may agree with in the margins, but for the really important things, there was not going to be agreement. The health and industry sides were going to disagree. Don't expect us to agree. Be prepared that there's a dispute here and you're going to have to decide which side you're going to come down on… I think we see it with most government, you know, they want it to be in partnerships and so the start of our argument was that partnerships are all a one-way street. You know the industry are wanting involvement in health policy. But I'm not being invited by* [large food retailer] *to decide on their pricing… policies at Christmas, you know. So, the partnership was very much a one-way street.* (CS14)

Table S6. Potential challenges to advance interventions

Key challenges	Details	Illustrative quotes
**1. Difficult to achieve or implement**	Participants noted likely difficulties in achieving or implementing some interventions. Most notably building coalitions and attaining consensus for international treaties (A13, A26,	*"…once governments have come together, it's in their hands, they have to make the consensus, WHO makes decisions by consensus, and that puts the industry champions, especially the United States, the Biden administration, into a very powerful position, because if you can be the only one who is against and then they have to find consensus there. So first we have to build a strong coalition. Before that we*

	A4, FPO4, CS6), reforming ITIAs and WTO rules (A2), or alternatives to neoliberalism and GDP growth (A16). Participants noted that UCIs would also need to be significantly denormalised through advocacy strategies for there to be sufficient political will to develop national policies, such as reforming political financing systems or tax laws due to political opposition (A23, A4, FPO4). Importantly such policy reforms may themselves be subject to industry sector influence. (A4). Moreover, many interventions require a significant amount of funding (FPO3, A1, A15), such as undertaking litigation against UCIs and defending against UCI litigation (A26), and interventions involving the WHO (that has a relatively small budget) (CS10).	*actually have to bring them together and then we have to have, I think the capacity of countries to overcome opposition from just a few very industry friendly countries."* (CS6)<br>"*A really high-level point. All this assumes… that the system can be changed. Need to consider the possibility that the…type* [of] *capitalism we have right now cannot be changed."* (A16-SN)<br>"*Resource mobilisation; HR; finance etc - very difficult to get; industry implements to make it difficult – this* [resource mobilisation] *is to ensure that governance & their mechanisms are strong enough.*" (FPO3-SN)<br>"*Something else in that direction is probably tax havens, tax avoidance, tax evasion. And we find in our field that some of the most environmentally destructive companies are those that are the most offshored companies. They are also the companies that have the largest global influence because they're multinationals, they're incorporated in multiple locations, and they have their money protected in different arrangements and that kind of stuff. That's more complicated because that's an international issue so that requires not domestic regulation but international regulation. And there's massive incentives not to. I think that's a meaningful leverage point, but I think it's probably extremely difficult to achieve because you're talking about not just shutting down a key element of the system for the for the health sector, but for all global sectors that manage their finances in that way."* (A4)<br>*"…funding parties and pet projects as well as campaigns… I think it depends on the political system. So somewhere like the US where effectively you know members of Congress have to generate tens of thousands of dollars every week to get re-elected every two years..."* (A23)<br>*"…the trade ministers, they're just so stuck in neoliberal ideology and also just getting trade agreements done for the sake of being shown to be doing things in signing agreements."* (A2)<br>*"…you get this support for neoliberalism, that you've actually got a whole system that's there about manufacturing consent for this whole system. And I think that's pretty powerful. And, you know, people take individual… health* [it] *is about behaviourism, rather about structural factors…"* (A9)<br>"*…*[the] *culmination of multistakeholderism which has been so much of the global debate for a long time and now we hear, you know the previous director General at WHO… was really strict on this stuff… Their* [WHO's] *remit is so broad they're being asked to do so much and save the world from pandemics and so on with like no money… it's often picked up on that WHO's annual budget is about the same, like same as a midsize city hospital in the US... It's nothing for what they're being us to do, so of course, they're desperate for the resources..."* (CS10)
**2. Partially formulated or implemented interventions**	Interventions need to be comprehensively designed and rigorously implemented or they risk being ineffective. For example, oversight, enforcement and sanction mechanisms are	"*Enforcement of regulations/laws/rules is imperative…*" (A16-SN)<br>*"Adequately resourced regulatory enforcement powers."* (A1-SN)<br>*"Many industry tactics/strategies are legal so need to look not just at the legal system."* (FPO1-SN)

	needed to be built into interventions, such as regulating public official interactions with UCIs, lobbying, and revolving doors (A1, A5, FPO2, A14, A25, A9, A15, CS18, A16, CS8, CS17, A19, CS15). For example, oversight, enforcement and sanction mechanisms are needed to be built into interventions, such as regulating public official interactions with UCIs, lobbying, and revolving doors. Transparency and disclosure mechanisms may also have limited effectiveness (A6, CS17, FPO4) if interactions with UCIs are still allowed (A25, A4). Such mechanisms could risk giving a false sense of effectiveness, (A25, A04, CS9), unless such interventions are paired with sanctions for violations (A25, CS19, A12). Participants suggested that interventions need to apply to all UCIs, not a selective few, (A16) and loopholes need to be closed (CS9, FPO4). Moreover, some forms of strategic litigation may be ineffective as UCI practices are legal (FPO1, CS3, CS17, FPO3). Lastly, adherence to existing interventions, such as Article 5.3, needs strengthening.	*"…if you allow any kind of grey areas then it is hard to prove, it is hard to enforce, it is hard to monitor it. It makes a lot of complications, especially with our modern digital marketing technologies, social networks…"* (A5)<br><br>"*We've also learned that, you know, there's more CoI* [rules] *than there used to be. But some of the CoI* [rules] *is pretty damn hopeless. And so, you get quite a lot of people who work for the industry who just don't declare their conflicts and so on…. All that said, would it be easy to implement for other* [unhealthy commodity] *industries?"* (CS17)<br><br>*"… if we know transparency in terms of conflict of interest,* [it] *probably doesn't do any good. It may make it worse. Education about conflict of interest doesn't do much good and somebody revealing that they have an association with some corporation, it doesn't take away their bias to actions, because… even though it's in the brain so much… they're not aware that they're biased…it leads to this false sense of things are OK because they've revealed their potential conflict of interest. But it's still a real conflict of interest, and there's something… in psychology called blind spot bias. People are biased and they don't know it. They're not aware of it… that happens to people who are appointed to government positions, for example, so that they are… being favourable towards corporations, towards industry. And they're not aware of it."* (A25)<br><br>*"…transparency, it's excellent. It's great, it's good, but it's not effective in many cases…*" (CS9)<br><br>"*This was my comment is around lobbying registers around transparency, accountability enforcement powers, cetera. And I know in our own experience here, we do have a lobbying register and we have standards in public office and since 2015 we have regulation of lobbying act. And you know it… kind of goes some of the way, it brings some transparency and for system. But even then, it's not enough and there's loopholes so it's creative compliance, ways of getting around us. We* [have] *seen only in the last two years a former minister of state jumping over to an industry organisation and didn't notify the standards in public office. It wasn't within the 12-month cooling off period. The public officer had mentioned a number of times where these designated public officials have breached the cooling off period, but they can't investigate, and they can't enforce. So essentially there's a law there and it's not effective, it has no teeth, and you know, that's problematic.*" (FPO2)<br><br>"*…there's a question about how effective lobbying registers are, and who counts as a lobbyist… we were working on international trade negotiations. A lot of it was done by law firms, for example, who have client privilege and have multiple relevant clients…. And does the law firm even reveal who its clients are because of* [lawyer-client] *privilege… I think the industry is probably several steps ahead of regulations like that in terms of working out kind of different ways to sort of meet people in with plausible deniability*" (A23)

		"So obviously lobbying isn't only an issue for alcohol policy or public health, and a lot of people think there are problems with the way lobbying goes on at the moment. There's a very less than half-hearted effort to introduce a lobbying register by [a particular] *government, which not many people think is effective at all…*" (FPO4)<br><br>"*…the reality is that none of the industry actions are illegal. Like, they make sure that they have actions that don't fall into illegal aspects because they know that's an easy path to go through to undermine action from their side."* (CS8)<br><br>*"Sometimes these things* [transparency mechanisms] *have a symbolic value beyond their actual kind of practical or material effects, because it signals that kind of direction of travel and its signals that kind of values* (sic) *that permeate society and shape the conduct of governance. So I'm certainly not saying these things* [transparency mechanisms] *were a bad idea or shouldn't be done, but they're as in of themselves, I think they're not an adequate solution to the issue, but it does set norms or it creates a precedent that you know government should be open and these things should be documented and… revealed, and the fact that there is freedom-of-information means they have to subvert it, right? And you know if there was no freedom-of-information, they would just carry on doing what they were doing behind closed doors."* (A23)
**3. Exploited or misused interventions**	Interventions could be exploited or misused; for example, participants warned that changes to investment practices, such as ESG investing, or SRI would need to be genuine and not co-opted by UCIs as a branding strategy (CS17, A26). Increasing democratic policymaking is important at all levels of governance, but it may risk enabling third-party groups representing industry to participate in policy discussions (A9). At the same time, advocates should not alienate all industry stakeholders, as they are not always aligned with each other (CS2). Additionally, UCIs can use some interventions, such as providing their data, to distract policymakers from their harmful practices (CS16).	*"Some* [investment companies], *they don't have a specific, some sort of written principle* [for ESG], *but you know, it's* [tobacco is] *excluded… the way the tobacco industry is fighting back is that, you know, they present themselves as… totally great ESG corporations. Even better than some, you know, I don't know, food industry. So, they get all these very good rankings, so… just to convince the investors to bring that money back, right. So that movement is already there so it's a matter of sort of leveraging on that. Making sure that they are making outright exclusions of tobacco, right? And in fact, there's a threat of them of bringing back tobacco now..."* (CS17)<br><br>*"How do we get to ESG investing? - tobacco put themselves at ESG corps; leveraging that movement*" (A26-SN)<br><br>"*…it's about introducing participatory governance that's genuine, and* [it] *isn't at the moment, as it is co-opted by industry front groups*" (A9).<br><br>*"…I want to think a bit more around who are the allies as well, because clearly industry can be very powerful and there are parts of the industry who actually have, maybe not all the time, but may have similar interests, in Scotland comes to mind where they were advocating for a minimum unit price and actually the hospitality industry: the hotel, the pubs themselves, were actually supportive. They're not friends, but they were allies of a particular policy, and so I think we need to think really. I'm not saying that they should necessarily have an open door, but I think we need to think really carefully about what our objective is by tarnishing all with the same brush and not thinking about the potential for these*

		other industries to have an aligned influence, something that actually reinforces the case, and I think this is something that we grappled with a lot, but aren't really nailing at the moment in this space." (CS2)<br><br>"*...when industry are* (sic) *controlling what data is released... there's no scrutiny. They're able to report on metrics that suit them. And we're not able to independently evaluate it. And the last one was the fig leaf policy. So, it's quite linked to the PPI* [public-private investment] *stuff, big distraction policies, essentially, that tie everyone up in. An example of this would be, we're expecting this mandatory reporting to potentially come in on food companies in the food strategy and there's a danger that we spend the next five years talking about metrics and who's going to report on what and how and to whom. And nobody is actually looking at what the companies are actually doing because we're just too busy talking about metrics and reporting*." (CS16)
**4. Interventions requires tailoring for context**	Although participants suggested that all of above interventions were needed in most countries, they noted that some specific countries or regions may need specific interventions depending on the UCI strategies to influence policy, and some interventions themselves should be tailored depending on the country or region, or for the particular industry they aim to address (CS20, A9, CS3, FPO3). Notably, participants highlighted the significant issue of corruption in some low- and middle-income countries and addressing it would be more appropriate as a first step in tackling UCI influence (A11, A13, FPO3, CS3). Other participants noted that due to political or social culture in less democratic counties, governments may be less willing to implement some interventions, such as regulating lobbying (CS13, A22, CS9, A9). Therefore, the specifics of each intervention may need adapting for what governments or other stakeholders are willing to implement. Participants also noted that interventions needed to be adapted for certain levels of governance (i.e., local, national, regional or international levels) (CS7, A11, CS12).	*"...another issue that stands for me, as someone that lives in a global south country, is the things that you are also addressing, you can adapt at least from my perspective to the reality of a global north or our global south country. So, I also want to highlight that. But one thing that also stands for me... depending on the reality of each country or region, probably I think could be like the thing [intervention or actions] for that specific country or specific for that region. And probably in other regions, those things could not be like so well developed or probably it will*." (CS20)<br><br>"*Best strategy and intervention would depend on the context for each country - would need to conduct a situational analysis. So would need a best practice or toolbox for best interventions to industry influence*" (FPO3-SN)<br><br>"*...the corruption we've had thus far, and I think in [my country's] political situation... politicians are very corrupt. So, as you know the corruption and the fact that we have easy access [to policymakers] together actually creates an even more viable platform for industry to actually walk right in*." (FPO3)<br><br>"*But obviously in countries like Mexico, Latin America and other countries is completely messed. Their lobbying is not even regulated. It's not even a thing. It doesn't even, you don't mention it.... you don't talk about this... So, it's a different level of action depending. It's not like one size fit all. So, as I'm saying we're dealing with people intimidating, extorting people, corruption very big problems of that* (sic), *so I think transparency, and as I said, watchdogs are important in those settings that are obviously... very different than* [in] *Europe..."* (CS3)<br><br>*"I think it's worth considering that because I think you know a lot of industry influence will border on corruption. So, you know indexes of corruption as showing that, the trend is getting worse rather than getting better."* (A9)

	Moreover, certain UCIs, such as food, may need unique interventions create fair competition (A4) and encourage the production and consumption of healthy alternatives (CS3), whilst others need to be ended such as tobacco (CS17).	*"… I agree that national and local level is, you know, where things should happen. And so… if international instruments…really empowered national and local governments somehow to be able to enforce the provisions of treaties and local laws, I think that would be really important."* (CS7)<br><br>*"If you were talking about one particular issue in one particular context, you could say, 'OK, I think the strategy for working with academics is XYZ, or the strategy for working with paediatricians is XYZ.' But you're looking at a complex web of relationships from the local and national level up to global so to tease those out, and to say which of those things is going to be most effective, no one I don't think would be able to give you that birds eye view. You can say 'this works here, that worked there, how do we scale?' And then there's all this question of scaling up and context."* (CS12)

Table S7. Key actions to help achieve interventions

Action category	Key action details	Illustrative quotes
**1. Coordinate and cooperate with stakeholders**	There should be efforts to create global collaborative networks to coordinate actions addressing UCI practices; for example, between academics, civil society, and policymakers (A6, CS12, A24, CS14, CS4, CS5) from different levels of government (CS6, A23, A6, CS2, CS8, CS13, CS5, CS9, A12, FPO3, CS12, A25, CS17, A16). These efforts could be facilitated by international governance organisations, such as the WHO (CS14).<br><br>Global collaborative networks could involve creating cohesive advocacy coalitions (A22, CS13, CS14, CS7, A25) that comprise a wide range of stakeholders (CS12, A24, CS14, CS4, CS5, A6, FPO3, CS1). Civil society groups could pool resources together (CS13, A25) and develop multistakeholder initiatives between governance institutions, academics, and civil society (CS13, CS18, A8, GGO1) to act as a counterweight to PPPs (GGO1).<br><br>Networks could advocate for broader political reforms, such as political transparency, democratic governance, and anti-corruption	*"…look at some tobacco control movements where… at that time* [initially] *they were working separately, but I think interacted synergistically together to* [achieve] *a positive outcome… Maybe they were not thinking that they're working together, but I think that this synergistic… effects did result or does result in a better outcome rather than having just one approach."* (A6)<br><br>*"There was* (sic) *no splits between… ourselves, the housing charities… other alcohol charities and other people who might be involved in this* [successful advocacy to help pass alcohol control policy]. *There was really no I think contrary voices on it… Price availability, marketing are* (sic) *central to this issue and there was no credible voice saying anything different, and so we worked quite hard to make sure that was…"* (CS14)<br><br>*"Cooperation between civil society groups interested in accountability of harmful industries to pressure for transparency."* (CS1-SN)<br><br>*"Need to join and unite different actors that align and work together e.g., academic and NGOs."* (CS13-SN)<br><br>*"Synergise siloed worked to prevent address corporate influence."* (A25-SN)<br><br>*"How do you get those things* [regulation] *to happen? Legislation is better that a voluntary agreement --> advocacy coalition."* (A22-SN)<br><br>*"[S]tronger (and well resourced) global coordination of independent efforts to tackle harmful practices of harmful product industries (with government and multilateral support and coherence)."* (CS2-SN)<br><br>*"…multistakeholder initiatives… it starts with ideas and to put out the idea that this* [industry practices] *isn't acceptable and then build support for that idea. So, for example, we did work at the community level in our food policy institute, training young people to do counter marketing of unhealthy food in their neighbourhoods and youth organisations. We developed some materials, we sent them around to legislators, and now a state senator has introduced a state law to limit predatory marketing of unhealthy food to children and that legislation creates*

	policies (A3, A25, CS1) and form alliances between governments of low- and middle-income countries to challenge large powerful corporations when developing policy (A1).<br><br>Networks could create international forums to discuss and share knowledge about policy solutions for UCIs (CS6, CS14) between policymakers from different countries (FPO4, CS6) especially including experts in economics and trade (A10). Stakeholders should try to find agreement and multilateral support on which industries require regulations (CS2). This could be achieved with government support for WHO guidance and advice (CS14).	*a forum for a state-wide debate among elected officials, civil society groups and the public... And I do see it as a multistage process and… we as public health researchers advocates, academics can bring those ideas into the public arena and look for actors who can put them forward into the policy, political, legislative arena.”* (A8)<br><br>*“More partnership between academics and institutions […] Academics keep institutions honest – collaborative centres → Close ties between them, can speak truth to power – act as a counterweight.”* (GGO1-SN)<br><br>*“I think if we're looking at the industry level that still fundamentally a sort of semi-health, unhealthy commodity industry focus, which isn't particularly helpful* [to achieve change]*... and that's probably the silo that we need to jump across, regardless of whether alcohol and tobacco talked to each other. I think if alcohol and tobacco start talking to each other* [only]*, they're still going to be talking in an echo chamber.”* (A3)<br><br>*“…we need something like that* [FCTC] *for alcohol… they create infrastructure and policy arenas where, these so-called ‘Conferences of Parties’ where you come together and can talk…”* (CS6)<br><br>*“… your policymakers are important, your decisionmakers are important… and I think civil society are critical together with media, so you need a very unique multidisciplinary approach […] that is really a critical part of implementation, no matter what your interventions are.”* (FPO3)<br><br>*“International dimension was NB* [for advocacy] *- national governments support WHO guidance and advice → there is a norm of this (attractive message)”* (CS14-SN)
**2. Invest in civil society**	Funding should be provided to civil society and grassroots organisations, such as through earmarked taxes (FPO4, FPO3, CS1, GGO1, CS11, CS2, CS4, CS5, A14, A26) to: expose industry practices (A15, A26); participate in technical policymaking processes (CS10); conduct public health campaigns though reframing strategies (CS5) showing that public health policies will work (CS1); create industry watchdogs (CS4); and ensure that civil society organisations do not need to rely on industry for funding (A6).<br><br>Participants suggested capacity building for civil society groups on (A2, A3, FPO4, CS12), how to engage in policy consultations and deliberations (GGO1) and the impact of new technologies, such as digital commerce, on public health (A2).	*“Funding of civil society organisations = strong CSOs* [civil society organisations]; *worked closely with gov; does not work with alcohol and health - need a funding mechanism* (sic)*.”* (FPO4-SN)<br><br>*“Even grassroots organisations are paid by industry and advocacy are paid by the EU – need to get funding for them but the question is how.”* (GGO1-SN)<br><br>*“Resource needs to be expanded for public health policy implementation/advocacy.”* (CS11-SN)<br><br>*“I mean, it sounds easy to do* [working together across unhealthy commodities], *but just from even where I'm sitting, which is in a research organisation, if you don't have funding, you don’t to work in a particular field, like alcohol, for example. I'm on the* [NGO] *board and I devote time and my organisation allows me to spend some time there, but we don't have any research funding for it, and it's really hard to get… alcohol, in particular… nobody wants to fund it”* (A14)<br><br>*“… we could do better if we had some more resources to expose to put pressure on government…”* (A15)<br><br>*“…funding support for civil society and technical decision-making committees, I think this is an area where, I mean, civil societies only recently got stuck in with some of these more technical committees and they tend to be very dominated by the industry interests… you get to that level of kind of decision-making, the real technical nitty gritty, and you realise, you know, that's actually where you need to be to make sure that the nuts and bolts*

		of regulations are neutral or not completely biased… so, support for civil society to engage at that level with that kind of level of expertise I think is really important." (CS10)<br><br>*"Some civil society orgs* (sic) *mobilise policymakers… mobilise public or other stakeholder groups eg doctors or patients etc – to sustain movement need funding - need victims for grassroot movements, need grass root organisations to convince policymakers"* (CS5-SN)<br><br>*"… there's* (sic) *lots of agencies* [philanthropic organisation] *who were starting to kind of get into the media….* [a big philanthropic organisation] *provided quite a lot of funding… So that helps to change social norms and soften the ground for policy reform because it talks about the problem and who's responsible for it. It takes it off the individual's responsibility, which is a really important shift, I think, for the public to get behind…* (CS1)<br><br>*"If you compare the history of policy on smoking compared with that on alcohol, when a lot of the policies on limiting smoking came in the 1970s there were very strong civil society organisations, which played a big role, bodies like ASH* [Action on Smoking and Health]. *And they were partly funded by government, they got quite generous funding and government worked closely with them and that doesn't happen at the moment with alcohol and health… it is important that, just to realise that government can't do all this on its own and some way needs to be found to strengthen the civil society organisations."* (FPO4)<br><br>*"Coordination to pool resources; clear objective and goals; how to get there?* (sic)*"* (CS13-SN)<br><br>"*I just added capacity building… because… there's a lot of… capacity around access to medicines and tobacco control and I'm guessing around… investment state dispute settlement, but like things like digital commerce and other things that I think* [are] *going to be more in focus that will have an impact on health. There isn't a lot of capacity or understanding or expertise, so… even myself, like I find it difficult to analyse like a trade context of e-commerce and understand how that's going to impact like rules around marketing… I think capacity building is definitely important."* (A2)
**3. Create a social movement**	A social movement should be developed between civil society, activists, and grassroots organisations to address all forms of industry influence on policy, including exposing (CS3, A25, CS5, CS4, A6, A26), denormalising (A6, CS5, A8, CS6, A10, CS19, CS14, A25), and showing similarities across UCI and their practices (CS12, A25, A23). Importantly, this could be achieved, for example, through victims of UCIs advocating for effective public health policy (CS5, A8, CS9, CS12). Civil society should participate in policymaking processes from local to global levels (A9, CS5, A18,	*"… need a creative movement to create a social mobilisation to whip up people in a genuine grassroot movement to create change and pressure policymakers to implement changes."* (CS5-SN)<br><br>*"[G]rassroots mobilisation - foster public 'outrage' and participation."* (A18-SN)<br><br>*"[V]iew change from policymakers that has come about from social mobilisation due to climate change."* (A4-SN)<br><br>*"…the other thing is making connections between these different silos* [unhealthy commodities public health advocacy], *and between these different sectors. So, there are lessons to learn from engagements with tobacco and alcohol, that would be really helpful in thinking through how to engage with government and industry around food. And that links with climate and that links with all these other industrial influences… we're quite scattered in our own response, and it would be really helpful to link up and to join the dots and to support one another*

	CS10), and activists should advocate for structural changes, such as alternative forms to economic growth (A4, A2).<br><br>Participants suggested various ways for civil society to be proactive; for example, organise protests (A25); create a complains systems for UCIs marketing (A24); leverage and work with existing fractions within UCIs (A1); develop campaigns on UCI harms using social media (CS13); and support political candidates who back democratic reforms and advocate for their election (CS13, CS5, A25).<br><br>Participants also suggested advocating to gain public support (CS1, A4, CS13) for policymaking reforms (A25, A1, A8, CS13, CS7), including democratising policymaking at multiple levels of governance (CS6, CS13, A22, A1, A9, CS8, A2, A10). Additionally, the public could volunteer with organisations that support democratic reforms, meet with local political representatives, and run for office (A25).	*because it's the same issues. And we're dealing with corporations that are incredibly powerful and well-resourced…"* (CS12)<br><br>*"… the other thing that many organisations* [civil society group] *and many of us have been involved in is also engaging with political parties… trying to influence their manifestos, trying to… get them to promise more progressive laws and this is again a part* [of]*… engaging with government that a lot of civil society is apprehensive in doing. But I think… that's something that can really help and we have seen currently."* (CS13).<br><br>*"…having grassroot community mobilisation and communities about tobacco use and harms… and then having policymaker advocacy pushing forward policy."* (A6)<br><br>*"… the actual victims are not willing to speak up… those are the actual people who suffer, and if we would take other examples where people suffer, they want… to speak up and they want to have justice* […] *we have those people excluded from the policy process while in many other similar cases… we could find HIV patients they demand what they need and, you know, they have their protest, their actions and they put that direct pressure on policymakers…. In order to have the impact that we need, and in order to have the actions that would be required to sustain this movement and to make it self-organised, this is basically impossible... if we cannot even have the victims if the victims themselves don't… speak up so all those grassroots movements. Well, they are important. But they are important to have them to show to the decisionmakers…."* (CS5)<br><br>*"… democratising global governance and giving public interest, civil society, and* [a] *much stronger role, for instance, in* [international] *trade discussions"* (A9)<br><br>"*The activism comes into place here and the social movements, that's… voting and running for office* [of] *people with ideology that is more progressive in terms of corporate regulation. So… both its social movement activism for laws and regulations and that would bring about regulations of lobbying, of the revolving door and election campaign finance… […] That's* [a] *pretty broad range of things, everything from contributing to not-for-profit groups who focus on these issues, to being a volunteer in organisations, working for organisations. Public health people, for example, could work in not-for-profit organisations that are focused on democratic reform to actually, you know, meeting with your legislators, mass campaigns of phone calls and social media and telephone calls and letters and emails. All that stuff to legislators to marches and protests."* (A25)<br><br>"*…keep engaging with the government and, you know, trying to, you know, sort of develop more pro-poor, more equitable policies so, you know, I mean so often as civil society… we are standing on the streets and sloganeering against the government, but also… have to be in committees and… try to also have a supportive role* [to government]*… and influence policies by actually writing them or… helping government to write them*" (CS13)<br><br>*"… advocates actually set up an alternative platform where people could submit complaints on their alcohol regulation because they just had a self-regulatory Portman type system over there that people weren't even submitting that much too, because nothing would come of it…. they received way more in-depth complaints about*

		a whole range of alcohol marketing practices, and it was an example of where civil society was actually setting up a… stronger system than the self-regulatory one by themselves and then they were able to publish evaluations of its effectiveness versus the industry one, which obviously involved collaborating with academics and that kind of thing." (A24)<br>*"…the whole thing of how democratic that inside those institutions are (sic)… that's the only thing that can be a counter to this…. reducing the harms in this… goes back to then democratisation, and as someone who has been involved in campaigns and movements and done so much of advocacy and a lot of street action and research…* [there are] *all different kinds… of ways to sort of just shake this web."* (CS13)<br>*"…in the energy, climate… space, and one of the most important strategies, as it were, to get a shift away from fossil fuels, is using the cleavage within the energy sector. So, within this sector, and so it was actually… U.S. policy and it was actually helping or bringing… electricity people into the policy discussions. They may not be 100% exactly what you would want, but they were, because they were supportive over progressive energy policy and were willing to speak against fossil fuels, so it's finding that cleavage within the industry."* (A1)
**4. Nurture leadership**	There is a need for policy leaders or champions in governance institutions (A16, CS17) to advance and support public health policies and policymaking reforms at national or international levels (A26, CS15) This would also help to ensure policy coherence across government departments (FPO4). However, participants also noted that the existence of policy leaders or champions at the right time is dependent on chance (A26, C17). Lastly, participants suggested that leaders who have personal experiences of UCIs' attempts to influence or deceived them should also speak out against UCIs (A6, CS5).	*"… you have to have strong champions at that* [international] *level and policies are made at that level."* (CS17)<br>*"Leadership is key. Leadership that understands conflict of interest and can act in the best interests of a population."* (A16-SN)<br>"[Article] *5.3 came out of a particular history and came of a particular point with all the FCTC and came at a particular point in time, and also, a particular happenstance. You know that you happen to have at the time a Director General of WHO who sort of got this issue […] we were talking about WHO and Brundtland, who was the terrific DG at the time, and the champions. And we need the champions at all levels. And some of them… we just strike lucky with… there happens to be somebody we didn't even know was going to be stronger in this area, who gets an influential role. We've got to be prepared for that. And we've also got to go out and find them."* (A26)<br>*"Political lead (leadership) helps to prevent policy incoherence between gov departments* (sic)*."* (FPO4-SN)<br>"*How do we get there!?* [effective health policies] *It's depressing to say we have to rely on luck! Is this is connected to leadership/policy champions?* (sic)*"* (CS15-SN)<br>*"So, like from my experience, I've seen cases when the tobacco industry promised, convinced the decisionmakers that things will go certain way. And then the decisionmakers realised that they were fooled, tricked lied to. And with sometimes based on that we were able to warn the others… that's what they've done. And again, the issue there is probably because those who were fooled don't want to speak up because they know with whom they are dealing with."* (CS5)
